# Methods to generate and validate a Pregnancy Register in the UK Clinical Practice Research Datalink primary care database

**DOI:** 10.1002/pds.4811

**Published:** 2019-06-13

**Authors:** Caroline Minassian, Rachael Williams, Wilhelmine H. Meeraus, Liam Smeeth, Oona M.R. Campbell, Sara L. Thomas

**Affiliations:** ^1^ Department of Non‐communicable Disease Epidemiology London School of Hygiene & Tropical Medicine London UK; ^2^ Clinical Practice Research Datalink Medicines and Healthcare Products Regulatory Agency London UK; ^3^ Department of Infectious Disease Epidemiology London School of Hygiene & Tropical Medicine London UK

**Keywords:** electronic health records, pharmacoepidemiology, pregnancy, pregnancy outcome, pregnancy trimesters, United Kingdom

## Abstract

**Purpose:**

Primary care databases are increasingly used for researching pregnancy, eg, the effects of maternal drug exposures. However, ascertaining pregnancies, their timing, and outcomes in these data is challenging. While individual studies have adopted different methods, no systematic approach to characterise all pregnancies in a primary care database has yet been published. Therefore, we developed a new algorithm to establish a Pregnancy Register in the UK Clinical Practice Research Datalink (CPRD) GOLD primary care database.

**Methods:**

We compiled over 4000 read and entity codes to identify pregnancy‐related records among women aged 11 to 49 years in CPRD GOLD. Codes were categorised by the stage or outcome of pregnancy to facilitate delineation of pregnancy episodes. We constructed hierarchical rule systems to handle information from multiple sources. We assessed the validity of the Register to identify pregnancy outcomes by comparing our results to linked hospitalisation records and Office for National Statistics population rates.

**Results:**

Our algorithm identified 5.8 million pregnancies among 2.4 million women (January 1987‐February 2018). We observed close agreement with hospitalisation data regarding completeness of pregnancy outcomes (91% sensitivity for deliveries and 77% for pregnancy losses) and their timing (median 0 days difference, interquartile range 0‐2 days). Miscarriage and prematurity rates were consistent with population figures, although termination and, to a lesser extent, live birth rates were underestimated in the Register.

**Conclusions:**

The Pregnancy Register offers huge research potential because of its large size, high completeness, and availability. Further validation work is underway to enhance this data resource and identify optimal approaches for its use.

**KEY POINTS**

- Large primary care databases are valuable sources of pregnancy data for studies of pregnancy.
- Identifying pregnancies, their timing, and outcomes in these databases presents challenges for researchers.
- We developed an algorithm to determine pregnancy episodes in the UK Clinical Practice Research Datalink (CPRD) GOLD primary care database.
- Our algorithm generated a Pregnancy Register comprising 5.8 million pregnancies among 2.4 million women from CPRD GOLD general practices spanning three decades.
- This data resource provides a useful tool to enhance CPRD‐based pregnancy research.

## 1 INTRODUCTION

Pregnant women are a key study population for many important health questions, including understanding the safety and effectiveness of drugs and vaccines given during pregnancy, effects of other in utero exposures on foetal outcomes, and long‐term sequelae of pregnancy complications. Electronic health primary care record (EHR) datasets contain a wealth of maternal and infant data, and their large size enables them to be used to assess rare exposures and outcomes in real‐world settings.^1^ However, ascertaining the timing and outcomes of pregnancies in such data presents challenges, since the start, end, and trimester dates of pregnancies are not systematically recorded.^2^ For studies investigating potential teratogenic risk factors, it is essential to estimate pregnancy start dates accurately, as this enables exposures during the first trimester, the critical period of organogenesis, to be identified.

Primary care datasets are increasingly used for pregnancy research, with researchers developing a multitude of methods to characterise pregnancies therein.^3^, ^4^, ^5^, ^6^, ^7^, ^8^, ^9^, ^10^, ^11^ These methods typically involve some of the following components: simple imputation^3^, ^5^ (subtracting a fixed duration from the pregnancy outcome date to derive the start date); mapping markers of pregnancy (diagnoses, appointments, or procedures indicative of a pregnancy) to pregnancy outcomes^7^, ^8^, ^12^; and utilising additional information in patient records to infer the start of pregnancy^3^, ^5^, ^6^, ^9^, ^13^ (eg, last menstrual period (LMP) dates, antenatal dating scans, or gestational age at birth). While some researchers attempt to characterise a variety of pregnancy outcomes,^7^, ^8^, ^13^ others restrict to live births.^3^, ^12^ Hence, the methods vary in their complexity, accuracy, and the situations in which they can be useful. Validation studies show that utilising multiple sources of information improves date estimation.^2^, ^14^ However, even the more complex approaches are limited by the exclusion of pregnancies with no recorded outcome,^7^, ^9^ outcomes with no earlier pregnancy marker,^9^, ^13^ or conflicting pregnancy records within the same woman.^7^, ^9^, ^13^ To date, there has been no published, systematic approach to characterise each documented pregnancy, including those with no recorded outcome, and to use all available pregnancy data in a primary care database.

The UK Clinical Practice Research Datalink GOLD primary care database (henceforth referred to as CPRD) is one of the largest, best‐established primary care databases for research. Our study aimed to develop, apply, and validate a new algorithm to identify pregnancies in CPRD, to facilitate and improve the quality of pregnancy research using CPRD. Building on our initial work to identify deliveries in CPRD,^15^ the CPRD Mother‐Baby link (restricted to live births) and other EHR‐based pregnancy algorithms,^2^, ^16^, ^17^ we sought to identify all documented pregnancies regardless of the completeness of recording or the type of outcome, to establish a Pregnancy Register in CPRD. Here, we describe our algorithm and the Pregnancy Register it generates, present our validation findings, and highlight key strengths and limitations of this data resource to enable researchers to understand its scope and optimise its use for pregnancy research.

## 2 METHODS

### 2.1 Data sources

CPRD is a database of routinely collected, anonymised primary care health records for over 15 million patients, representing the UK population in age, sex, and ethnicity.^18^ CPRD comprises records of consultations, diagnoses and symptoms, prescriptions, tests, referrals to and feedback from secondary care, health‐related behaviours, and all additional care administered as part of routine general practice. In the United Kingdom, general practitioners (GPs) are the main point of contact for nonemergency health issues, including pregnancy. Thus, CPRD is a rich source of pregnancy data relating to antenatal and postnatal care and pregnancy outcomes. A practice‐specific family number enables mother‐infant pairs to be algorithmically linked (the CPRD Mother‐Baby link). Patients from 56% of CPRD GOLD practices can be linked to additional datasets, including Hospital Episode Statistics (HES), which comprises records of all patient care delivered by NHS hospitals in England, including maternity data. We used CPRD GOLD primary care data to generate the Pregnancy Register and linked HES data to validate it. The International Scientific Advisory Committee (ISAC)‐approved protocol for this study (ref 11_058) is provided in the Supporting information.

### 2.2 Generating pregnancy code lists

CPRD GOLD codes clinical events using the hierarchical read classification system. GPs may also record additional, structured data using entity codes. To maximise ascertainment of pregnancy data, we generated lists of read and entity codes relating to pregnancy. We identified an extensive set of pregnancy‐related terms from relevant chapters of the read hierarchy, used in combination with wildcards, to identify potentially relevant read codes. We then compared these with existing code lists^7^ to identify additional codes. We identified relevant entity codes from the “child health surveillance” and “maternity” chapters. We excluded irrelevant codes and categorised our final selection of codes in 21 nonmutually exclusive categories shown with examples in Table 1. Our complete categorised code lists of 4200 read codes and 37 entity codes and details of how the algorithm uses the codes and accompanying data fields in each category are provided in Data S2 to S4.

**Table 1:** Pregnancy code categories and example codes

Pregnancy Code Category	Number of Read Codes^a^	Example Read Code	Description
Antenatal	1446	62…00	Patient pregnant
Late pregnancy (≤3 wk before delivery)	35	L281.00	Premature rupture of membranes
Third trimester	47	62N8.00	A/N 32 week examination
Delivery	1030	L20..11	Spontaneous vaginal delivery
Stillbirth	29	Q4z..15	Stillbirth NEC
Ectopic	28	L03..00	Ectopic pregnancy
TOP	148	L052.11	Medical abortion—complete
Miscarriage	70	L04..00	Spontaneous abortion
Probable TOP	5	L05..12	Termination of pregnancy
Molar pregnancy	11	L002.00	Complete hydatidiform mole
Unspecified pregnancy loss	96	L0z..00	Pregnancy with abortive outcome NOS
Blighted ovum	2	L010.00	Blighted ovum
Postnatal (≤8 wk after delivery)	902	62S7.00	Postnatal examination normal
Other postnatal^b^	97	E204.11	Postnatal depression
Preterm	28	L142.11	Premature delivery
Postterm	18	L150.00	Postterm pregnancy
Multiple	96	L210.00	Twin pregnancy
LMP	1	1513.00	Last menstrual period—first day
EDD	6	1514.12	Estimated date of delivery
EDC	3	Z22C500	Estimated date of conception
Pregnancy related (timing uncertain)^b^	371	L12..00	Hypertension complicating pregnancy/childbirth/puerperium

Abbreviations: A/N = antenatal; EDC = estimated date of conception; EDD = estimated date of delivery; LMP, last menstrual period; NEC = not elsewhere classified; NOS = not otherwise specified; TOP, termination of pregnancy.

a Categories are not mutually exclusive; hence, codes may appear in more than one category.

b Codes in these categories are not used to determine pregnancy start and end dates due to uncertainty around which stage of pregnancy or the postnatal period they refer to.

### 2.3 Study population

We identified all female patients aged 11 to 49 years from CPRD GOLD practices during the period between January 1, 1987, and February 28, 2018, with individual‐level research quality data and with a pregnancy code in their primary care records. We extracted all their pregnancy records and additional data on timing and gestational age at birth from live‐born infants identified in the Mother‐Baby link. We applied no further restrictions. Hence, records relating to time periods before patients joined a practice or before practices' data were deemed to be of a research quality standard (indicated by the practice up‐to‐standard date) were included. This enabled us to generate a complete pregnancy profile for each patient.

### 2.4 Summary of the pregnancy algorithm

The pregnancy algorithm used all available pregnancy data (from read and entity codes) to determine the timing of pregnancy (start, end, and trimester dates), the outcome (live birth, stillbirth, or early pregnancy loss), and additional details including whether a pregnancy was preterm, postterm, or multiple. The algorithm began by classifying each patient's pregnancy outcome records into distinct pregnancy episodes (combining multiple records relating to the same outcome) and estimating pregnancy end dates. Delivery records were considered separately from early pregnancy loss records. In keeping with UK clinical practice,^19^ we considered the onset of the LMP to be the pregnancy start.

**Figure 1:**
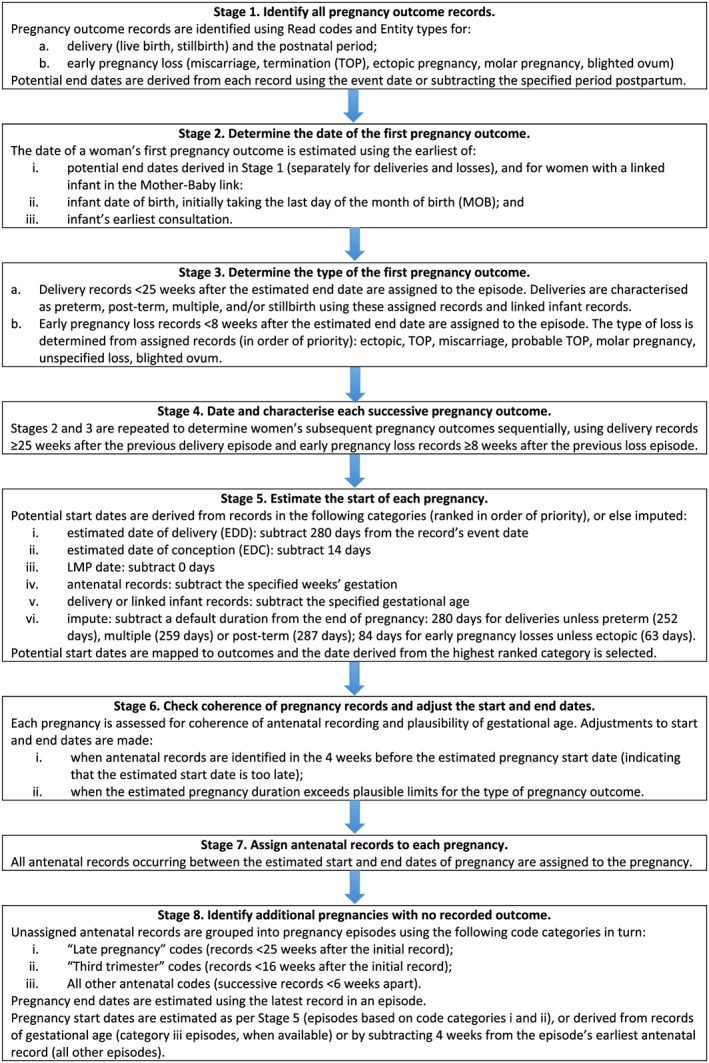
Stages of the Clinical Practice Research Datalink (CPRD) GOLD pregnancy algorithm [Colour figure can be viewed at wileyonlinelibrary.com]

We derived pregnancy start dates from multiple read and entity codes in the following order of priority: (a) estimated date of delivery (EDD), (b) estimated date of conception (EDC), (c) LMP, (d) antenatal records indicating gestational age, and (e) gestational age at birth (from maternal and infant records). EDD was preferred over EDC and LMP as these codes were considered more likely to derive from an antenatal ultrasound scan, and hence to be more reliable, than a record of LMP. Indeed, codes relating to a scan were used preferentially when available. Codes indicating gestational age during pregnancy or at delivery often specified a range rather than a precise number of weeks, hence these were positioned further down the hierarchy. Additionally, because codes indicating gestational age at delivery were considered more prone to delayed recording, which could result in a delayed estimated start date, these were used only in the absence of codes in other categories. In the absence of such records, we applied a fixed duration, consistent with the type of pregnancy, to impute the start date. We estimated the timing of trimesters to be LMP onset to 13 completed weeks for the first trimester, weeks 14 to 26 for the second, and week 27 to delivery for the third. The entity codes and associated data fields used to derive pregnancy start and end dates are shown in Table 2. Characteristics of each pregnancy episode, including the type of delivery or pregnancy loss (when recorded), were determined from the pregnancy codes, which the algorithm assigned to the episode. Figure 1 illustrates the eight stages of our algorithm. Full details are provided in the Supporting information.

**Table 2:** Entity codes used to derive pregnancy start and end dates

Entity Code	Description	Estimation of Pregnancy Date
To estimate the start of pregnancy		
60	Ante‐natal booking	Event date minus the number of weeks specified in the relevant data field^a^ (allowing a maximum of 42 wk).
61	Ante‐natal consultation	
154	Alpha fetoprotein	
119	Gestation—maternity outcome	Estimated date of delivery (derived by the algorithm) minus the number of weeks specified in data 1 (allowing a maximum of 42 wk).
120	Gestational age of baby	
129	Pregnancy dates	Expected delivery date (in data 2) minus 280 d.
284	Maternity ultra sound scan	Expected delivery date (in data 8) minus 280 d. If data 8 is missing, use event date minus the number of weeks specified in data 2 (allowing a maximum of 42 wk).
To estimate the end of pregnancy		
35	Hearing (6 wk)	Event date minus 42 d.
80	Muscle tone for 6 wk (CHS)	
84	Vision CHS 6 wk	
63^b^	CHS examination	If CHS stage (in data 2) = birth, use event date. If CHS stage = 6 wk, use event date minus 42 d.
69	Postnatal examination	Event date minus the number of days or weeks specified in data 2 (allowing a maximum of 56 d or 8 wk).
150	Postnatal visit	
78	Stages of labour	Event date
93	Delivery details	
112	CHS Apgar score at 1 min	
115	Delivery details (CHS)	
119	Gestation—maternity outcome	
120	Gestational age of baby	
126	Maternity infant details	
128	Perineum	
144	Maternity outcome placenta	
145	CHS Apgar score at 5 min	
100	Perinatal problems	Event date minus 7 d.
114^c^	Pregnancy outcome	Discharge date (in data 1) minus 2 d.

Abbreviation: CHS, Child Health Surveillance.

a Data 1 (entity codes 60 and 61), data 8 (entity code 154).

b Used only if CHS stage specifies birth or 6 weeks.

c Used to estimate either a delivery date or an early pregnancy loss date (depending on the outcome specified in the record). All other entity codes are used to estimate delivery dates only.

The Pregnancy Register lists and describes all pregnancies identified in CPRD GOLD by our algorithm. Each record represents a unique pregnancy episode. There may be more than one episode per woman. For pregnancies resulting in live births, patient identifiers of babies identified in the Mother‐Baby link are provided. Full descriptions of the Pregnancy Register variables are shown in Table S1.

### 2.5 Validation methods

#### 2.5.1 Internal validation

We assessed the validity of our algorithm to identify pregnancy outcomes occurring in hospital by comparison with linked HES Admitted Patient Care data (HES APC, henceforth referred to as HES).

##### Data sources and study population

We included women aged 11 to 49 years, who were registered with an HES‐linked CPRD GOLD practice from England and eligible for linkage. Women were required to have a pregnancy outcome recorded in the Pregnancy Register (January 2016 prototype) or in HES (Set 13) between April 1, 1997, and December 31, 2015. Both data sources were concurrent during this period.

HES diagnoses are based on the International Classification of Disease clinical coding system (ICD‐10), and procedures are coded using the OPCS Classification of Interventions and Procedures (OPCS‐4). Deliveries were determined from the HES maternity file, and additional data on pregnancy outcomes were extracted using OPCS codes for end‐of‐pregnancy or postnatal procedures and ICD codes for early pregnancy loss and combined into pregnancy episodes. Full details of the approach and codes used to determine pregnancy episodes in HES are provided in the Supporting information.

##### Analysis

We compared the occurrence of deliveries and early pregnancy losses in HES to those captured in the Register and assessed agreement. Follow‐up for pregnancy outcomes in both data sources began at the latest of: the patient's 11th birthday, the date they joined the practice, the practice up‐to‐standard date, or the start of HES coverage (April 1, 1997). Follow‐up ended at the earliest of: their 49th birthday, the date they left the practice or died, the practice's last collection date, or the end of CPRD coverage (December 31, 2015). We considered a match to occur if a woman's pregnancy outcome dates in CPRD and HES were less than 12 weeks apart. We chose 12 weeks to allow for potential errors in date estimation in the Register, assuming that pregnancy outcomes recorded in the two data sources within 12 weeks apart represent the same event. We calculated the potential positive predictive value (PPV) (recognising that not all women deliver in hospital), completeness of recording, and accuracy of timing of pregnancy outcomes in the Register, using HES as the reference standard. Additionally, for matched deliveries (those in both data sources and less than 12 weeks apart), we assessed concordance on gestational age (completed weeks).

**Figure 2:**
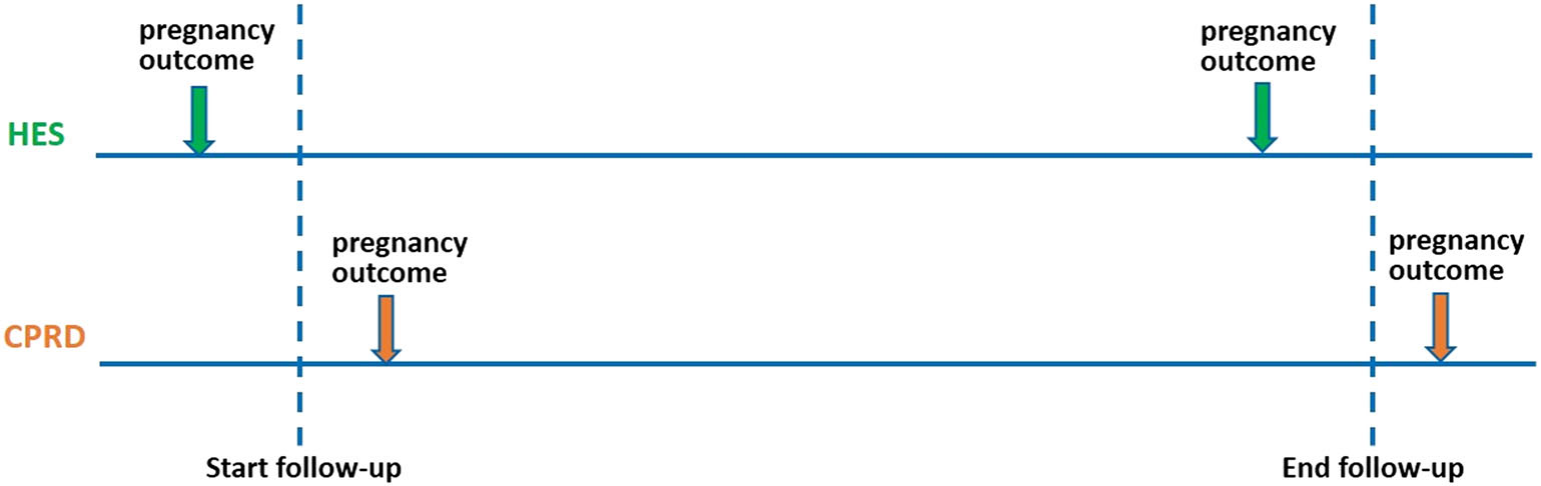
Potential impact of delayed recording of hospital pregnancies in Clinical Practice Research Datalink (CPRD) on agreement between data sources [Colour figure can be viewed at wileyonlinelibrary.com]

**Figure 3:**
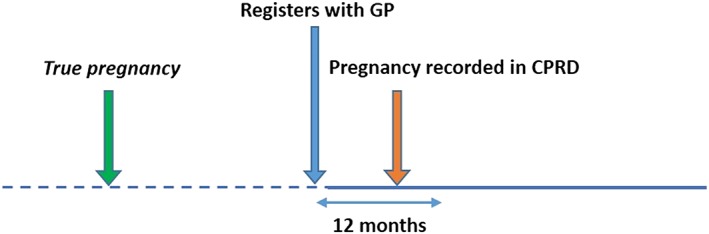
Retrospective recording of historical pregnancies in Clinical Practice Research Datalink (CPRD) [Colour figure can be viewed at wileyonlinelibrary.com]

We explored reasons for incomplete matching in sensitivity analyses. First, to allow for possible delays in GPs recording pregnancy outcomes occurring in hospital, we excluded Register‐recorded pregnancies in the first 6 months of follow‐up (when calculating PPV), and HES‐recorded pregnancies in the last 6 months of follow‐up (when calculating sensitivity). As shown in Figure 2, including these pregnancies potentially underestimates agreement between the two data sources. Second, to allow for possible retrospective recording of past pregnancies soon after a patient joins a practice^20^ (Figure 3), we excluded pregnancies recorded in the Register during the first year of registration, as historical pregnancies are unlikely to be captured in HES.

#### 2.5.2 External validation

We assessed the validity of the Pregnancy Register estimates of live birth, miscarriage, termination, and prematurity rates by comparing with national vital statistics and published estimates.

##### Data sources and study population

To ensure comparability with external estimates regarding age, geographic region, and time, we restricted our study population to women aged 15 to 44 years, registered with CPRD GOLD practices in England and Wales from 2010 to 2015. Patient follow‐up began at the latest of: their 15th birthday, the date they joined the practice (plus 1 year, to avoid ascertainment of historical pregnancies), the practice up‐to‐standard date, or January 1 of the calendar year. Follow‐up ended at the earliest of their 44th birthday, the date they left the practice or died, the practice's last data collection date, or December 31 of the calendar year. This external validation used a later version of the Pregnancy Register (February 2018).

##### Analysis

We estimated live birth and termination rates in 2015, defined as the number of live birth deliveries, and the number of terminations, each per 1000 women‐years. We chose person‐time as a denominator (rather than the mid‐year number of women) to allow for the dynamic nature of our cohort. In secondary analyses, we expanded our definition of termination to include “probable termination” and “unspecified loss” (see example codes in Table 1). We conducted sensitivity analyses, extending follow‐up to include patients' first year of registration, to increase ascertainment of live births and terminations among women who joined a practice while pregnant. We estimated the miscarriage rate over a 5‐year period (2010‐2015), defined as the number of miscarriages divided by the total number of pregnancies with known outcomes. In a secondary analysis, we expanded our definition of miscarriage to include “unspecified loss.” We estimated the prematurity rate in 2015, defined as the number of preterm deliveries (less than 37 weeks of gestation) per thousand births (live births and stillbirths). The primary analysis included as preterm those deliveries with a preterm flag in the Register (based on evidence in read and entity codes assigned to the pregnancy). In a secondary analysis, we expanded our preterm definition to include additional deliveries with estimated gestational age of less than 259 days (less than 37 weeks).

## 3 RESULTS

### 3.1 Pregnancy Register profile

The February 2018 version of the Pregnancy Register included 5 824 381 pregnancies among 2 438 493 women overall, of which 1 503 685 (pregnancies) occurred during up‐to‐standard follow‐up, among 765 867 women who had been registered at a practice for at least 1 year. This latter subset comprised 291 826 early pregnancy losses and 833 197 deliveries, of which 543 866 (65.27%) had a Mother‐Baby link, and 24 038 (2.89%) were characterised as preterm, 57 557 (6.91%) postterm, and 6790 (0.81%) multiple births. Additionally, 378 662 pregnancies with no recorded outcome were identified, of which 33.20% were potentially ongoing when follow‐up was censored (the earliest antenatal record in the episode was less than 38 weeks before the patient left the practice or the practice last collection date). The distributions of pregnancies among women and by outcome are shown in Tables 3 and 4, respectively.

**Table 3:** Pregnancy distribution in women aged 11 to 49 years in the Clinical Practice Research Datalink (CPRD) GOLD Pregnancy Register from January 1, 1987, to February 28, 2018

Number of Pregnancies^a^ (n)	Women with n Pregnancies, N (%)	
1	383 196	(50.03)
2	198 859	(25.97)
3	94 859	(12.39)
4	46 561	(6.08)
5	22 002	(2.87)
6	10 568	(1.38)
7	4982	(0.65)
8	2465	(0.32)
9	1195	(0.16)
10+	1180	(0.15)
Total women	765 867	(100)

a Refers to pregnancies of women registered for at least 1 year at a practice contributing research quality data.

**Table 4:** Distribution of pregnancy outcomes in the CPRD GOLD Pregnancy Register from January 1, 1987, to February 28, 2018

Pregnancy Outcome	Frequency (%)	
Live birth	786 647	(52.31)
Stillbirth	3515	(0.23)
Live birth and stillbirth	32	(0.00)
Miscarriage	132 998	(8.84)
Termination (TOP)	18 052	(1.20)
Probable TOP	114 268	(7.60)
Ectopic pregnancy	11 056	(0.74)
Molar pregnancy	935	(0.06)
Blighted ovum	533	(0.04)
Unspecified loss	13 984	(0.93)
Delivery based on a third trimester code	19 915	(1.32)
Delivery based on a late pregnancy code	23 088	(1.54)
Outcome unknown	378 662	(25.18)
Total pregnancies^a^	1 503 685	(100)

Abbreviations: CPRD, Clinical Practice Research Datalink; TOP, termination of pregnancy.

a Refers to pregnancies of women registered for at least 1 year at a practice contributing research quality data.

Women had a median of one pregnancy (interquartile range [IQR] 1‐2 pregnancies) and less than 1% had more than seven pregnancies. The median gestational age at delivery was 280 days (IQR 273‐280), and median 84 days (IQR 84‐84) for early pregnancy losses. For pregnancies with known outcomes, the median gestation at the first antenatal record was 53 days (IQR 40‐72). Pregnancy start dates were imputed for 42% of pregnancies with known outcomes, though for relatively fewer deliveries (30%) than for early pregnancy losses (76%). Pregnancies whose start dates were not imputed had shorter gestations (median 278 days (IQR 266‐285) for deliveries, 76 days (IQR 56‐98) for early pregnancy losses). Overall, 14.0% of women had pregnancy episodes that appeared to overlap (238,242 pregnancies).

### 3.2 Validation results

#### 3.2.1 Internal validation

##### Deliveries

**Figure 4:**
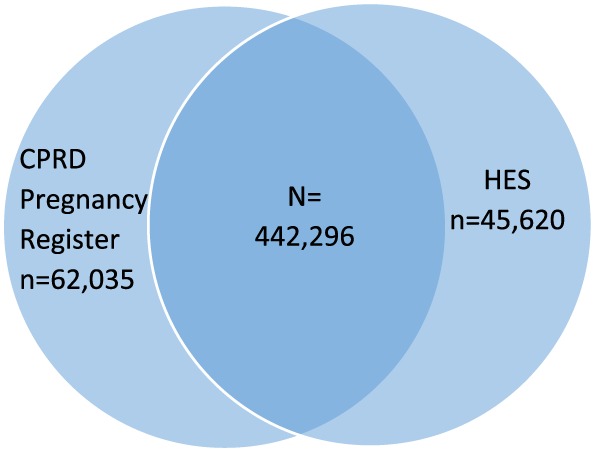
Venn diagram of deliveries (live births and stillbirths) identified in Clinical Practice Research Datalink (CPRD) GOLD and Hospital Episode Statistics (HES) [Colour figure can be viewed at wileyonlinelibrary.com]

Using the linked data, we identified 386 955 women with a delivery recorded in either the Pregnancy Register (504 331 deliveries) or in HES (487 916 deliveries) during the study period. Overall, 442 296 matches were identified (deliveries captured in both data sources less than 12 weeks apart) among 328 450 women (84.9% of the delivery cohort) (Figure 4). A large majority of Register deliveries had a corresponding HES delivery (potential PPV 87.7%). The remaining 12.3% (n = 62 035) had no HES match. Similarly, most HES deliveries had a corresponding Register delivery (sensitivity 90.7%). The remaining 9.3% (n = 45 620) had no Register match.

A total of 50.9% of matched deliveries had the same date of delivery in each data source, and 94.9% differed by no more than 2 days. The median days' difference between matched delivery dates (Register date minus HES date) was 0 days (IQR −2 to 0 days). A record of gestational age in the HES maternity file was present for 304 982 (69.0%) matched deliveries. The median weeks' difference in gestational age for matched delivery pairs (Register minus HES) was 0 weeks (IQR 0‐0 weeks); the mean difference was 0.13 weeks.

##### Early pregnancy losses

**Figure 5:**
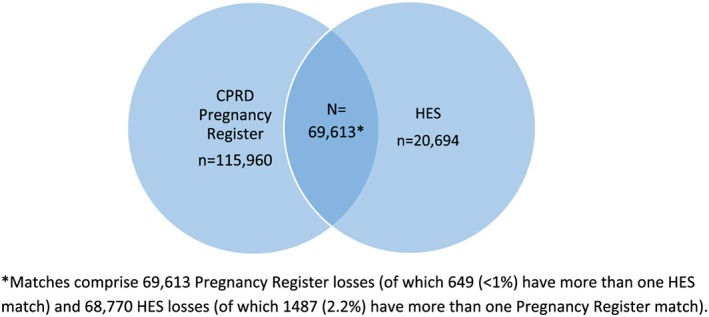
Venn diagram of early pregnancy losses identified in Clinical Practice Research Datalink (CPRD) GOLD and Hospital Episode Statistics (HES) [Colour figure can be viewed at wileyonlinelibrary.com]

We identified 160 839 women with an early pregnancy loss recorded in the Pregnancy Register (185 573 losses) or in HES (89 464) during the study period. Overall, 69 613 matches were identified among 60 115 women (37.4% of the pregnancy loss cohort) (Figure 5); 37.5% of Register losses had an HES match. A higher proportion of HES losses had a Register match (sensitivity 76.9%); 115 960 Register losses and 20 694 HES losses had no match.

A total of 47.4% of matched pregnancy losses had the same date in each data source, and 85.1% differed by no more than 2 days. The median days' difference between matched pregnancy loss dates (Register date minus HES date) was 0 days, IQR: −1 to 0 days.

##### Sensitivity analyses

Excluding 60 429 Register deliveries and 27 587 Register losses within the first 6 months of follow‐up led to modest increases in potential PPV (88.9% for deliveries, 38.2% for losses). Similarly, excluding 100 804 Register deliveries and 34 067 Register losses in the first year of registration marginally increased potential PPV (88.3% and 38.4%, respectively). Excluding 52 164 HES deliveries and 7 887 HES losses within the last 6 months of follow‐up increased sensitivity to 93.2% for deliveries and 78.6% for losses.

### 3.3 External validation

Our external validation findings comparing pregnancy outcome rates in the Pregnancy Register with external estimates from Office for National Statistics (ONS) and other published evidence are shown in Table 5. Our algorithm identified 21 806 live births and 317 terminations (3489 including “probable terminations” and “unspecified losses”) among 599 493 women (472 283 women‐years) in 2015, generating lower rates of live birth (46.17) and terminations (0.67‐7.39) per 1000 women‐years compared with external estimates (62.32 live births^21^ and 16.59 terminations^22^ per 1000 women); 271 090 pregnancies with known outcomes occurred in the Register between 2010 and 2015, of which 33 722 ended in miscarriage (35 763 including “unspecified losses”), producing miscarriage rates of 12.44% to 13.19% consistent with external estimates (12.5% to 20%).^23^, ^24^ Of the 21 892 Register deliveries in 2015, 782 were flagged as preterm by the algorithm, and a further 1059 were identified as preterm based on gestational age, increasing the prematurity rate to 8.41%, consistent with ONS (7.91%).^25^

**Table 5:** Comparison of pregnancy outcome rates in the CPRD GOLD Pregnancy Register with external estimates

Pregnancy Outcome, Calendar Period	CPRD GOLD Pregnancy Register Rate (95% CI)	External Comparison Rate (Data Source)
Live birth rate^a^, 2015		
Primary analysis	46.17 (45.56‐46.79)	62.32 (ONS)
Sensitivity analysis (includes first year of registration)	51.55 (50.94‐52.16)	
Miscarriage rate^b^, 2010‐2015		
Primary analysis (miscarriage)	12.44 (12.32‐12.56)	12.5 (NHS), 11‐20 (Ammon Avalos et al, 2012)
Secondary analysis (miscarriage and unspecified loss)	13.19 (13.07‐13.32)	
Termination rate^a^, 2015		
Primary analysis (TOP)	0.67 (0.60‐0.75)	16.59 (DHSC)
Secondary analysis 1 (TOP and probable TOP)	6.98 (6.74‐7.22)	
Secondary analysis 2 (TOP, probable TOP and unspecified loss)	7.39 (7.14‐7.64)	
Sensitivity analysis (includes first year of registration)	0.72 (0.65‐0.80)	
Prematurity rate^b^, 2015		
Primary analysis (preterm flag^c^)	3.57 (3.33‐3.83)	7.91 (ONS)
Secondary analysis (preterm flag^c^ and estimated gestational age < 259 d)	8.41 (8.05‐8.78)	

Abbreviations: CI, confidence interval; DHSC, Department of Health and Social Care; NHS, National Health Service; ONS, Office for National Statistics; TOP, termination of pregnancy.

a Live birth and termination rates are per 1000 women‐years (Pregnancy Register), per 1000 women (external).

b Miscarriage rates are per 100 pregnancies, prematurity rates are per 100 deliveries (live birth and stillbirth).

c Based on evidence in read and entity codes assigned to the pregnancy.

## 4 DISCUSSION

Our goal was to establish a useful research tool for researchers planning to use CPRD data for pregnancy studies. The resulting Pregnancy Register, comprising more than 5.8 million pregnancies (more than 1.5 million meeting patient and practice‐level data quality standards) among 2.4 million women, spanning three decades, is the first of its kind in a UK primary care database. Our assessment of the internal and external validity of our algorithm to determine pregnancy episodes demonstrates high validity in identifying and dating hospital deliveries (91% sensitivity, 95% with date agreement within 2 days), and 77% sensitivity for hospital‐based early pregnancy losses (85% with date agreement within 2 days). Miscarriage rates in the Pregnancy Register of 12% to 13% compared favourably with estimates from external sources, whereas lower rates were observed for terminations and live births. Prematurity rates were lower when based solely on preterm evidence in pregnancy codes but improved markedly when gestational age was taken into account (8%). Overall, the scale and scope of pregnancies captured in the Register and our validation findings demonstrate the potential of this data resource to enhance future CPRD‐based pregnancy research.

Our algorithm has several advantages over previous pregnancy‐identification approaches in CPRD. A key strength is our use of all available pregnancy data across the entire patient record, including additional clinical details (entity codes) recorded in structured data areas to characterise and estimate the timing of each pregnancy. In contrast to previous approaches, we impose no restrictions regarding the completeness^13^ or timing^7^ of recording. Hence, pregnancy episodes based on a single antenatal code and historical pregnancies occurring prior to patient registration are included in the Register. This allows us to capture all documented pregnancy episodes, including pregnancies with no recorded outcome, which recent approaches have not addressed.^7^, ^13^ While such pregnancy episodes can be challenging to interpret, ignoring them is potentially more problematic and could lead to bias, particularly for studies requiring a denominator of pregnant women such as vaccine uptake studies. When outcomes are recorded, we use all available records to classify the episode, including differentiating between induced and spontaneous abortions whenever possible, rather than combining these episodes in a single “abortion” category as a recent approach has done.^13^

A key feature of our algorithm is it avoids preferentially selecting one type of pregnancy outcome over another. Because delivery episodes are generated separately from early pregnancy loss episodes, we are able to distinguish distinct pregnancy episodes for each type of outcome from multiple records corresponding to the same pregnancy, without choosing between outcomes when a patient has successive records of both (eg, a delivery code followed by a miscarriage code). By contrast, previous approaches discard pregnancy outcome records occurring within a pre‐specified time period after a patient's previous outcome, disregarding the type of outcome specified in the records, which could potentially result in incomplete ascertainment of distinct pregnancy episodes of different types.^13^

For studies of live birth pregnancies, a clear advantage of our approach is linkage between the Register and the Mother‐Baby link, which enables outcomes recorded in infant records, such as congenital malformations, to be assessed. However, 31% of Register deliveries (excluding stillbirths and deliveries based on late pregnancy or third trimester codes) had no linked infant. Because the Mother‐Baby linkage is based on GP practice and requires that the estimated delivery date and infant birth date (derived from month of birth) are less than or equal to 60 days apart, incomplete linkage may occur if the infant is not registered at the mother's practice during the data collection period, or due to imprecision in delivery or birth date estimates resulting in more than 60 days difference. The inclusion of some historical deliveries or possible misclassification of some stillbirths as live births in the Register may also partly explain the incomplete linkage.

A further strength of our approach is its transparency. We provide full details of our algorithm stages in the Supporting information, including our complete categorised pregnancy code lists (read and entity codes). This enables researchers planning to use the Pregnancy Register to understand its scope and assess its applicability for their particular study questions. The provision of a data field “start source” in the Register enables researchers to determine how pregnancy start dates were derived, eg, through imputation or from the available data.

When assessing capture of HES deliveries in the Register, high concordance was found. Some of the 9% of HES deliveries with no Register match could be deliveries with feedback from secondary care that were not coded by the practice but simply documented in free text fields of the practice software (which are unavailable to researchers). The incomplete concordance we observed between deliveries in the Register and those in HES may partly be due to approximately 4% of deliveries occurring at home, in private hospitals or in hospitals outside England.^26^ Indeed, we would not expect 100% PPV for Register deliveries for this reason. Nevertheless, this is unlikely to explain the remaining 8% of Register deliveries with no HES match.

A surprising finding from our external validation is the lower live birth rate in the Register compared with ONS in 2015, despite the high sensitivity we observed in comparison with HES. It is possible that HES‐linked practices have different completeness of recording of live births than practices not linked to HES. Because our Register‐ONS comparison includes practices that are not HES‐linked, the lower Register live birth rate could be due to these practices missing some live births. Any such difference in completeness of recording of live births in HES‐linked practices versus practices not linked to HES may limit the generalisability of our internal validation findings to the whole Pregnancy Register.

Other potential reasons for incomplete matching of pregnancy outcomes in the Register and HES include possible reporting delays, or retrospective recording of past pregnancies in primary care. Our sensitivity analysis findings provide some support for this: excluding Register and HES pregnancies in the first and last 6 months of follow‐up and Register pregnancies recorded in the first year of registration with a practice, marginally improved the algorithm performance, both in terms of PPV and completeness for deliveries and early pregnancy losses. Incomplete capture or misclassification of pregnancy outcomes in either data source could also partly explain the lack of concordance. The median gestation at the first antenatal record among Register pregnancies was 7.6 weeks, which suggests that a proportion of pregnancies resulting in early miscarriage would not be identified in the Register.

The higher agreement we observed with HES for deliveries compared with early pregnancy losses could partly be due to women who give birth having increased opportunity for their pregnancy outcomes to be recorded through GP consultations with their babies, than women whose pregnancies do not yield an infant. Furthermore, because of difficulties distinguishing between types of early pregnancy loss in HES, our analysis includes a heterogeneous group of outcomes (miscarriages, terminations, ectopic pregnancies etc.) However, we would expect miscarriages to be better recorded than terminations due to a substantial proportion of terminations being carried out in specialist clinics outside of NHS hospitals. Our validation findings of similar miscarriage rates yet lower termination rates in the Register compared with external sources reflect this.

There are limitations to our algorithm that are important to consider when using the Register. While our algorithm maximises all available pregnancy data, the reverse side of this approach is that some identified pregnancies included in the Register may represent historical events discussed during a consultation and recorded with the current date. However, researchers can apply restrictions on data occurring within patient registration and practice‐level up‐to‐standard follow‐up if required for their particular study question. Our validation analyses restricted to pregnancies occurring during up‐to‐standard follow‐up, hence the findings are not necessarily generalisable to pregnancies occurring before the practices' data were deemed up‐to‐standard.

Uncovering pregnancy episodes with no discernible outcome is also a consequence of our approach to maximise completeness of pregnancy ascertainment. Overall, these “outcome unknown” pregnancy episodes comprise 25% of all research‐quality pregnancies in the Register (among women registered for at least 1 year, at an up‐to‐standard practice). This is consistent with more than 20% of these types of pregnancies identified in an earlier pregnancy record mapping algorithm using CPRD.^8^ Our findings suggest that one‐third of these pregnancy episodes with unknown outcome are potentially ongoing pregnancies; however, the remainder are more difficult to interpret. Such episodes may occur for a number of reasons, for example, some may represent undocumented miscarriages requiring no medical intervention or early pregnancy losses with feedback from secondary care that were not captured in the coded data. Variability in the PPV of certain codes used to identify pregnancies, for example, codes which relate to pregnancy planning rather than a current pregnancy, might also explain some of these outcome unknown episodes. Comparing the estimated pregnancy dates in the Register with the date follow‐up is censored can help researchers decide whether a pregnancy is potentially or unlikely to be ongoing.

A further caveat of our approach is that it yields some patients' pregnancy episodes that appear to overlap. While some overlapping episodes may be an artefact of GP recording practices (for example, an apparent pregnancy loss nested within a delivery episode may represent a threatened miscarriage recorded as a “miscarriage,” culminating in a later delivery), others may arise from errors in date estimation. While these episodes also present interpretational challenges, we do not attempt to resolve them. Instead, such episodes are flagged as “conflicts” in the Register, allowing researchers to judge how best to handle them in the context of their own study questions. Characterising these “outcome unknown” and overlapping pregnancy episodes and exploring potential reasons for their occurrence are key areas of ongoing validation work to improve the Pregnancy Register.

## 5 CONCLUSIONS

We have described our approach to identifying and characterising pregnancies in the CPRD GOLD database and establishing a new data resource for pregnancy studies using CPRD data. The Pregnancy Register is available to researchers alongside existing CPRD GOLD datasets, upon receipt of ISAC approval of a study protocol. Further work to refine the Register and to extend it to data contributed by practices using EMIS software (CPRD Aurum) is ongoing.

## ETHICS STATEMENT

Ethics approval for this study was obtained from the Independent Scientific Advisory Committee for Medicines and Healthcare products Regulatory Agency database research (ref 11_058) and the London School of Hygiene and Tropical Medicine Ethics Committee (ref 16287).

## CONFLICT OF INTEREST

W. M. is currently an employee of GlaxoSmithKline (GSK) and holds GSK stocks/shares. At the time the work described in this manuscript was conducted, W. M. was an employee of the Clinical Practice Research Datalink, Medicines and Healthcare Products Regulatory Agency. The rest of the authors declare no conflict of interest.

## Supporting information

Table S1. CPRD GOLD Pregnancy Register field descriptions.

Table S2: How the algorithm uses pregnancy codes, dates and additional data fields within each code category.

Data S3: Supporting Information

Data S4: Supporting Information

Data S5: Supporting Information

Data S6: Supporting Information

Data S7: Supporting Information

Data S8: Supporting Information
