# Supplementary material for: Methods to generate and validate a Pregnancy Register in the UK Clinical Practice Research Datalink primary care database
Source: Pharmacoepidemiol Drug Saf. 2019 Jun 13;28(7):923–33. doi: 10.1002/pds.4811 (PMC6618019; doi:10.1002/pds.4811)
Supplement: Supplementary file 1 — Table S1. CPRD GOLD Pregnancy Register field descriptions. [file PDS-28-923-s001.docx]

S1-Table. CPRD GOLD Pregnancy Register field descriptions.

| *Field name* | *Description* | *Type* | *Format* |
| --- | --- | --- | --- |
| patid | Encrypted unique patient identifier | INTEGER | 20 |
| pregid | Unique identifier of the pregnancy episode | INTEGER | 20 |
| mblbabies | Number of babies the pregnancy is linked to in the MBL | INTEGER | 2 |
| babypatid^1^ | Encrypted unique patient identifier (linked baby) | INTEGER | 20 |
| babymob | Baby’s month of birth as recorded in the baby’s medical record | INTEGER | 2 |
| babyyob | Baby’s year of birth as recorded in the baby’s medical record | INTEGER | 4 |
| totalpregs | Total number of identified pregnancy episodes (per woman) | INTEGER | 2 |
| pregnumber | Pregnancy episode number (per woman) | INTEGER | 2 |
| pregstart | Estimated start date of pregnancy | DATE | dd/mm/yyyy |
| firstantenatal | Date of earliest antenatal record within the pregnancy | DATE | dd/mm/yyyy |
| startsource | Data source used to estimate pregnancy start date: 1 = Imputed^2^, 2 = EDD, 3 = LMP, 4 = Gestational age at birth, 5 = Gestational age from antenatal record, 6 = EDC | INTEGER | 1 |
| startadj | Flag to indicate whether the pregnancy start date has been adjusted: 0 = Not adjusted, 1 = Due to antenatal records in the preceding 4 weeks, 2 = Due to specific conflicts between the estimated pregnancy duration and records indicating gestational age at birth (live births and stillbirths only), 3 = Both | INTEGER | 1 |
| secondtrim^3^ | Estimated start date of second trimester | DATE | dd/mm/yyyy |
| thirdtrim^3^ | Estimated start date of third trimester | DATE | dd/mm/yyyy |
| pregend | Estimated end date of pregnancy. NB: For pregnancies with unknown outcome, the date of the latest antenatal record in the pregnancy episode is provided. | DATE | dd/mm/yyyy |
| endsource | Data source used to estimate pregnancy end date: 1 = Delivery record, 2 = Postnatal record in the mother’s medical record, 3 = Discharge date relating to a delivery, 4 = Baby’s (month and) year of birth as recorded in the baby’s medical record, 5 = Postnatal record in the baby’s medical record, 6 = First consultation in the baby’s medical record. Only completed for live births and stillbirths. | INTEGER | 1 |
| endadj | Flag to indicate whether the pregnancy end date has been adjusted: 0 = Not adjusted, 1 = Due to specific conflicts between the estimated pregnancy duration and records indicating gestational age, 2 = Due to prior adjustments to the start date, 3 = Both. Missing for deliveries based on late pregnancy records^4^. | INTEGER | 1 |
| gestdays | Estimated duration of pregnancy episode in days (calculated as pregend minus pregstart) | INTEGER | 3 |
| matage | Mother’s age at end of pregnancy (years) | INTEGER | 3 |
| outcome | Outcome of pregnancy: 1 = Live birth, 2 = Stillbirth, 3 = 1 and 2, 4 = Miscarriage, 5 = TOP, 6 = Probable TOP, 7 = Ectopic, 8 = Molar, 9 = Blighted ovum, 10 = Unspecified loss, 11 = Delivery based on a third trimester pregnancy record, 12 = Delivery based on a late pregnancy record^4^, 13 = Outcome unknown | INTEGER | 1 |
| preterm_ev | Flag to indicate evidence of a premature delivery: 1=preterm, 0=no evidence of preterm, 9=not applicable (outcome not a delivery) | INTEGER | 1 |
| postterm_ev | Flag to indicate evidence of a post-term delivery: 1=post-term, 0=no evidence of post-term, 9=not applicable (outcome not a delivery) | INTEGER | 1 |
| multiple_ev | Flag to indicate evidence of a multiple pregnancy: 1=multiple, 0=no evidence of multiple. Missing for pregnancy losses. | INTEGER | 1 |
| conflict | Flag to indicate whether the pregnancy episode overlaps with another episode (per woman): 1=overlapping, 0= non-overlapping | INTEGER | 1 |

MBL=Mother-baby link; EDD=estimated date of delivery; LMP= last menstrual period; EDC=estimated date of conception; TOP=termination of pregnancy

^1^ A single babypatid is provided. For multiple pregnancies resulting in >1 liveborn infant (when mblbabies>1), additional babypatids may be retrieved from the MBL.

^2^ For “Outcome unknown” pregnancies, the imputed start date is obtained by subtracting 4 weeks from the earliest antenatal record in the episode.

^3^ The timing of trimesters is estimated using a common convention: first trimester (first day of LMP [pregstart] to 13 completed weeks), second (weeks 14 to 26), and third (week 27 to delivery [pregend]).

^4^ Late pregnancy records refer to the period up to 3 weeks before delivery, e.g. “Baby overdue”.
