# Supplementary material for: Methods to generate and validate a Pregnancy Register in the UK Clinical Practice Research Datalink primary care database
Source: Pharmacoepidemiol Drug Saf. 2019 Jun 13;28(7):923–33. doi: 10.1002/pds.4811 (PMC6618019; doi:10.1002/pds.4811)
Supplement: Supplementary file 2 — Table S2: How the algorithm uses pregnancy codes, dates and additional data fields within each code category. [file PDS-28-923-s002.docx]

S2-Table: How the algorithm uses pregnancy codes, dates and additional data fields within each code category.

| **Pregnancy code category** | **Code type and additional data fields used** | | **Patient records queried** | |
| --- | --- | --- | --- | --- |
|  | Read code | Entity code | Mother | Infant† |
| antenatal (any stage, from early to late pregnancy)* | Use event date | Entity codes 60 (data1=weeks gestation), 61 (data1=weeks), 88, 104, 105, 107, 116, 129, 154 (data8=weeks), 209, 229 (if data1=21, 30 or 31, i.e. positive pregnancy test), 284 (data2=estimated size in weeks), 320, and 487. Use event date for all. | ✓ | N/A |
| late pregnancy (codes relating to the period ≤3 weeks before delivery)* | Use event date | Entity codes 60 (data1=weeks gestation), 61 (data1=weeks), 154 (data8=weeks), and 284 (data2= estimated size in weeks) if specified number of weeks ≥39. Use event date for all. | ✓ | N/A |
| third trimester* | Use event date | Entity codes 60 (data1=weeks gestation), 61 (data1=weeks), 154 (data8=weeks), and 284 (data2= estimated size in weeks) if specified number of weeks ≥27. Use event date for all. | ✓ | N/A |
| delivery* | Use event date | Entity codes 78, 93, 112, 115, 119 (data1=weeks), 120 (data1=weeks), 126, 128, 144, and 145: use event date.  Entity code 114 (if data2=1 or 2, i.e. outcome is delivery): use “Discharge Date” in data1 minus 2 days. | ✓ | ✓ (codes specifying gestational age at birth) |
| stillbirth | Use event date | Entity code 114 (if data2=2, i.e. outcome is stillbirth): use “Discharge Date” in data1 minus 2 days.  Entity code 126 (if data7=2, i.e. outcome is stillbirth): use event date. | ✓ | N/A |
| ectopic pregnancy | Use event date | N/A | ✓ | N/A |
| miscarriage | Use event date | Entity code 114 (if data2=3, i.e. outcome is miscarriage): use “Discharge Date” in data1 minus 2 days. | ✓ | N/A |
| termination of pregnancy (TOP) | Use event date | N/A | ✓ | N/A |
| unspecified pregnancy loss | Use event date | N/A | ✓ | N/A |
| molar pregnancy | Use event date | N/A | ✓ | N/A |
| blighted ovum | Use event date | N/A | ✓ | N/A |
| postnatal (up to 8 weeks post-delivery) | Use event date minus the specified number of days/weeks postnatal (14 days when number not specified). | Entity codes 35, 63 (if data2=1 or 2, i.e. examination at birth or at 6 weeks), 69 (data2=weeks postnatal), 80, 84, and 150 (data2=days postnatal). Use event date minus the specified number of days/weeks postnatal.  Entity code 100: use event date minus 7 days | ✓ | ✓ (codes specifying the number of days/weeks postnatal) |
| preterm* | Use event date | Entity codes 119 (data1=weeks) and 120 (data1=weeks) if specified number of weeks <37. Use event date for all. | ✓ | ✓ |
| post-term* | Use event date | Entity codes 60 (data1=weeks gestation), 61 (data1=weeks), 119 (data1=weeks), 120 (data1=weeks), 154 (data8=weeks), and 284 (data2= estimated size in weeks) if specified number of weeks ≥41. Use event date for all. | ✓ | ✓ |
| multiple pregnancy | Use event date | Entity code 120 (if data3=2, 3, or 4, i.e. twin, triplet or multiple): use event date. | ✓ | ✓ |
| last menstrual period (LMP) – 1st day | Use event date (if not equal to system date). | N/A | ✓ | N/A |
| estimated date of delivery (EDD) | Use event date (if not equal to system date). | Entity code 129: use date in data2. Entity code 284: use date in data8. | ✓ | N/A |
| estimated date of conception (EDC) | Use event date (if not equal to system date). | N/A | ✓ | N/A |

Event date is the date associated with the event, as entered by the GP. System date is the date the event was entered into the Vision software system. N/A=not applicable

* Note that some of these Read and Entity codes specify gestational age, and hence are used to derive pregnancy start dates.

† Identified in the CPRD GOLD Mother-Baby link.
