# Supplementary material for: Methods to generate and validate a Pregnancy Register in the UK Clinical Practice Research Datalink primary care database
Source: Pharmacoepidemiol Drug Saf. 2019 Jun 13;28(7):923–33. doi: 10.1002/pds.4811 (PMC6618019; doi:10.1002/pds.4811)
Supplement: Supplementary file 6 — Data S6: Supporting Information [file PDS-28-923-s006.docx]

**Identifying pregnancy outcomes in linked Hospital Episode Statistics (HES) Admitted Patient Care (APC) data to validate the Pregnancy Register.**

Deliveries

Deliveries in HES were determined from:

- the HES Maternity file, using the data fields in Table 1 below.
- the HES Procedures file, using OPCS codes for delivery-related procedures (S5-Table).

The HES Maternity file includes records relating to episodes of admitted patient care during which a baby was delivered. There may be multiple records per episode of care, often relating to multiple births. A unique identifier for each episode of care in HES (epikey) enables linkage between HES APC data files. Records in the HES Maternity file were merged with the HES Episodes file to determine the type of episode of care (general, delivery or birth).

A summary Maternity record for each episode of patient care was created, retaining all available information from relevant data fields (those relating to a delivery) across an episode. The relevant data fields are described in Table 1.

Table 1.Data fields used to identify deliveries in the HES Maternity File

| **HES data field** | **Description** |
| --- | --- |
| epitype | Type of episode of care (general, delivery, birth etc.) |
| epistart | Episode start date |
| epiend | Episode end date |
| antedur* | Antenatal days of stay |
| postdur* | Postnatal days of stay |
| birordr | Birth order |
| birweit | Baby’s birth weight |
| biresus | Resuscitation method used on the baby |
| birstat | Whether the baby was born alive or dead |
| delonset | Method used to induce labour |
| delinten | Intended type of delivery place |
| delplac | Actual type of delivery place |
| delchang | Reason for changing the type of delivery place |
| delmeth | Method used to deliver baby |
| delprean | Anaesthetic or analgesic administered during labour and delivery |
| delposan | Anaesthetic or analgesic administered after delivery |
| delstat | Status of person conducting the delivery |
| gestat | Length of gestation (number of completed weeks) |
| matage | Mother’s age at delivery |
| sexbaby | Sex of baby |
| numbaby | Number of babies delivered at the end of a single pregnancy |

* Derived fields: antedur=number of days from epistart to delivery; postdur=number of days from delivery to epiend.

Potential delivery dates were derived for each summary Maternity record, using the antedur and postdur fields (when not missing) by adding the specified number of antenatal days of stay to epistart, or by subtracting the specified number of postnatal days of stay from epiend. The number of delivery-related fields with a valid value was determined for each summary Maternity record. At least 2 valid fields was considered sufficient evidence for a delivery.

Records of delivery-related procedures were identified from the HES procedures file using OPCS codes (categories 1 and 2, S5-Table). A summary Procedure record for each episode of patient care was created, retaining all delivery-related procedures occurring during the episode. The date of each procedure was determined from the data field “evdate”. When evdate was missing, epistart was used.

The summary Maternity records were merged with the summary Procedure records to create a combined (Maternity+Procedure) record per episode of care. Records with sufficient evidence for a delivery (≥2 valid delivery-related fields) or a delivery-related procedure were retained.

Estimated delivery dates were derived for each retained record, taking the earliest of the potential delivery dates (based on antedur or postdur) and procedure dates. For records with no potential delivery dates or procedures, epistart was used.

Delivery episodes were created by grouping together records relating to the same delivery (records with an estimated delivery date <25 weeks after the initial record were deemed to relate to the same delivery).

Early pregnancy losses

Early pregnancy losses in HES were determined from:

- the HES diagnosis file, using all ICD-10 codes for early pregnancy loss (S5-Table) across an episode of care. The start date of the episode of care (epistart) was used to date the pregnancy loss.
- the HES procedures file, using OPCS codes for early pregnancy loss procedures (category 3, S5-Table). As for deliveries, the date of the procedure (evdate) was used (when not missing), otherwise epistart was used.

Early pregnancy loss episodes were created by grouping together diagnosis and procedure records relating to the same pregnancy loss (records occurring <8 weeks after the initial record were deemed to relate to the same episode).
