# Supplementary material for: Methods to generate and validate a Pregnancy Register in the UK Clinical Practice Research Datalink primary care database
Source: Pharmacoepidemiol Drug Saf. 2019 Jun 13;28(7):923–33. doi: 10.1002/pds.4811 (PMC6618019; doi:10.1002/pds.4811)
Supplement: Supplementary file 7 — Data S7: Supporting Information [file PDS-28-923-s007.doc]

# ISAC APPLICATION FORM:

# PROTOCOLS FOR RESEARCH USING THE GPRD DATA

| ISAC use only:  Protocol Number  Date submitted | Team 1  11_058  9 May 2011 | **IMPORTANT**  **If you have any queries, please contact ISAC Secretariat:** ISAC[@gprd.com](mailto:Annalisa.Rubino@gprd.com) |
| --- | --- | --- |

| 1. Study Title   Extending the GPRD Mother Baby Link |
| --- |
| 1. Does this protocol describe a purely observational study using GPRD data (this may include the review of anonymised free text)?   Yes No |
| 1. Does this protocol also seek access to data held under the GPRD Data Linkage Scheme?   Yes  No |
| 1. If you are seeking access to data held under the GPRD Data Linkage Scheme, please select the source(s) of linked data being requested.   Hospital Episode Statistics  Cancer Registry Data  MINAP  ONS Mortality Data  Index of Multiple Deprivation/ Townsend Score  GPRD Mother Baby Link  Other: (please specify) |
| 1. If you are seeking access to data held under the GPRD Data Linkage Scheme, have you already discussed your request with a member of the Research team?   Yes  No*  **Please contact the GPRD Research Team on +44 (20) 3080 6383 or email* [*admin@grpd.com*](mailto:admin@grpd.com) *to discuss your requirements* ***before*** *submitting your application.* |
| 1. Does this protocol involve requesting any additional information from GPs?   Yes*  No  * Please indicate what will be required:  Completion of questionnaires by the GP*^ψ^* Yes No  Provision of anonymised records (e.g. hospital discharge summaries) Yes No  Other (please describe)  *^ψ^ Any questionnaire for completion by GPs needs to be approved by ISAC before being sent out for completion.* |

**GUIDANCE ON ANSWERING QUESTIONS 4-6:**

**These questions must be completed by all applicants. You should note the following:**

**(i) If you have answered NO to question 2, may need to seek separate ethics approval from an NHS Research Ethics Committee for this study. The ISAC will provide advice on whether this may be needed.**

**(ii) If you have answered YES answered to question 2 above and you will be using data obtained from the GPRD Group at the MHRA, this study does not require separate ethics approval from an NHS Research Ethics Committee.**

**If you will be using data obtained from EPIC, you will need to consult the data provider regarding their arrangements for obtaining ethics approval for the study.**

**NB:** Answering YES to question 2 means that the answers to questions 7-9 should all be NO. If any of the answers below are YES please review your answer to question 2 as it should be NO.

| 1. Has this protocol been peer reviewed by another Committee?   Yes* No  ** Please state in your protocol the name of the reviewing Committee(s) and provide an outline of the review process and outcome.* |
| --- |
| 1. Does the study involve linking to patient *identifiable* data from other sources?   Yes No |
| 1. Does this study require contact with patients in order for them to complete a questionnaire?   Yes No |
| 1. Does this study require contact with patients in order to collect a sample?   Yes* No  ** Please state what will be collected* |
| 1. Type of Study *(please tick one box below)*   Adverse Drug Reaction Drug Use  Disease Epidemiology Pharmacoeconomic  Drug Effectiveness  Other |
| 1. Data source *(please tick one box below)*   GPRD, MHRA:  Sponsor has on-line access  Purchase of ad hoc dataset  Commissioned study  Other  *(please specify)* GPRD |
| 1. Financial Sponsor of study   Pharmaceutical Industry *(please specify)*        Academia*(please specify)*  Government / NHS *(please specify)*        None  Other *(please specify)* |
| 1. This study is intended for:   Publication in peer reviewed journals  Presentation at scientific conference  Presentation at company/institutional meetings  Other |
| 1. Principal Investigator (full name, job title, organisation & e-mail address for correspondence regarding this protocol)   Rachael Boggon, Research Statistician, GPRD, rachael.boggon@mhra.gsi.gov.uk |
| 1. Affiliation (full address)   GPRD, MHRA, 151 Buckingham Palace Road, Victoria, London, SW1W 9SZ |
| 1. Type of Institution (please tick one box below)   Academia Research Service Provider  Pharmaceutical Industry  NHS  Government Departments  Others |

| 1. Experience/expertise available   Please complete the following questions to indicate the experience/expertise available within the team of researchers actively involved in the proposed research, including analysis of data and interpretation of results |
| --- |
| Previous GPRD Studies Publications using GPRD data  None  1-3  > 3 |
| Yes No  Is statistical expertise available within the research team?  *If yes, please outline level of experience* *GPRD Research Team*  Is experience of handling large data sets (>1 million records)  available within the research team?  *If yes, please outline level of experience* *GPRD Research Team*  Is UK primary care experience available within the research team?  *If yes, please outline level of experience* *GPRD Research Team* |
| 1. Other collaborators (if applicable: *please list names and affiliations of all collaborators*)   Tim Williams & Tjeerd van Staa (GPRD), Preeti Dataa-Nemdharry, Nirupa Dattani & Alison Macfarlane (City University), Helen Dolk (Ulster University), Kate Fleming (Nottingham University), Rosemary Tate (Brighton & Sussex Medical School) |
| 1. Protocol’s Author (if different from PI) |

**Protocol content checklist**

In order to help ensure that protocols submitted for review contain adequate information for protocol evaluation, ISAC have produced instructions on the content of protocols for research using GPRD data. These instructions are available on the GPRD website ([www.gprd.com/ISAC](http://www.gprd.com/ISAC)). All protocols using GPRD data which are submitted for review by ISAC must contain information on the areas detailed in the instructions. IF you do not feel that a specific area required by ISAC is relevant for your protocol, you will need to justify this decision to ISAC.

Applicants must complete the checklist below to confirm that the protocol being submitted includes all the areas required by ISAC, or to provide justification where a required area is not considered to be relevant for a specific protocol. Protocols will not be circulated to ISAC for review until the checklist has been completed by the applicant.

**Please note, your protocol will be returned to you if you do not complete this checklist, or if you answer ‘no’ and fail to include justification for the omission of any required area.**

|  | **Included in protocol?** | |  |
| --- | --- | --- | --- |
| **Required area** | **Yes** | **No** | **If no, reason for omission** |
| ***Lay Summary (max.200 words)*** |  |  |  |
| ***Background*** |  |  |  |
| ***Objective, specific aims and rationale*** |  |  |  |
| ***Study Type***  *Hypothesis Generating*  *Hypothesis Testing* |  |  | **This study is about data linkage, not hypotheses generation or testing** |
| ***Study Design*** |  |  | **As above** |
| ***Sample size/ power calculation***  ***(Please provide detailed justification of***  ***sample size in the protocol)*** |  |  | **As above** |
| ***Study population***  ***(including estimate of expected number of***  ***relevant patients in the GPRD)*** |  |  |  |
| ***Selection of comparison group(s) or controls*** |  |  |  |
| ***Exposures, outcomes and covariates*** |  |  |  |
| ***Data/ Statistical Analysis***  *Hypothesis Generating*  *Hypothesis Testing* |  |  | **As above** |
| ***Patient/ user group involvement ^†^*** |  |  |  |
| ***Limitations of the study design, data sources***  ***and analytic methods*** |  |  |  |
| ***Plans for disseminating and communicating study results*** |  |  |  |

***^†^ It is expected that many studies will benefit from the involvement of patient or user groups in their planning and refinement, and/or in the interpretation of the results and plans for further work. This is particularly, but not exclusively true of studies with interests in the impact on quality of life. Please indicate whether or not you intend to engage patients in any of the ways mentioned above.***

*ISAC strongly recommends that researchers using GPRD consider registering as a NRR data provider in order that others engaged in research within the UK can be made aware of current works. The* ***National Research Register (NRR)*** *is a register of ongoing and recently completed research projects funded by, or of interest to, the United Kingdom's National Health Service. Information on the NRR is available on* [*www.nrr.nhs.uk*](http://www.nrr.nhs.uk/) *.*

***Please Note: Registration with the NRR is entirely voluntary and will not replace information on ISAC approved protocols that are published in summary minutes or in the ISAC annual report****.*

**EXTENDING THE MOTHER BABY LINK**

**PROTOCOL**

**LAY SUMMARY**

The GPRD Mother Baby Link currently utilises information available within the database to identify mother and baby pairs. This allows researchers to study individuals within family units (e.g. whether taking a specified drug during pregnancy is associated with certain outcomes in the baby). However, there is the possibility to extend the scope from a mother to live baby link based purely on GPRD data, to a full pregnancy register incorporating data from HES and ONS mortality data.

**BACKGROUND**

The ability to study individuals within family units in epidemiology is very useful. As well as being able to look at familial aspects of disease, increased focus is being placed on the importance of in-utero exposures with respect to conditions manifested in the offspring. The best examples are studies into the teratogenic effects of drugs, which are hard to investigate using traditional pre-marking methodologies such as RCTs for obvious ethical reasons.

In GPRD there is a practice-specific family number that can be used to identify people within the same family. This is primarily based on residence, but can be edited in Vision where members of different families live at the same address. The existence of this variable allows us to link members of families together, and particularly mothers with their children. Furthermore, because both pregnancy and delivery information is recorded during the long follow up time within GPRD, it is possible to follow some children registered on GPRD from conception rather than from birth. This means the GPRD can be used to undertake the sort of studies that require information in all three trimesters.

To help users identify the linked mothers and children GPRD provide a Mother – Baby linked list. This list was first produced in 2004, extended in 2005, and was updated in 2010 to run using GPRD Gold data. The list is currently created on a monthly basis with new Read codes and entity types added. Now that the link is running efficiently on GPRD Gold data, there is the possibility to refine and extend the scope from a mother to live baby link based purely on GPRD data, to a full pregnancy register incorporating data from HES and ONS mortality data.

**OBJECTIVES, AIMS AND RATIONALE**

There are two main objectives of this study:

1. To refine the mother baby link, evaluating the opportunities brought by linked HES and ONS data.
2. To extend the mother baby link to a full pregnancy register including pregnancies resulting in termination, miscarriage and stillbirth.

**STUDY POPULATION**

Patients will be identified independently in the GPRD, HES and ONS data on the basis of dataset specific codes and variables related to pregnancy and its outcomes:

GPRD Read codes (clinical, referral and test files)

Entity types[[1]](#footnote-2) (clinical and test files)

Prescription exemption (patient file)

Consultation type (consultation file)

Referral specialism (referral file)

HES Episode type (delivery and birth files)

ICD codes

OPCS procedure codes

ONS ICD codes

The mother’s age at each estimated delivery date will be calculated as the difference between the year of the estimated delivery date and the mother’s birth year. Delivery records will be restricted to mothers between the ages of 12 and 49.

All GPRD registered acceptable patients born after 1986 will be considered for inclusion as children in the mother baby link.

Data will be utilised from the full time period available for each data source (i.e. 1987 to 2011 for GPRD, and 1997 to 2010 for HES/ONS) and across all practices (although HES/ONS data will only be available for practices consenting to take part in the linkage program, currently 224 in England).

The last monthly version of the existing Mother Baby Link (March 2011) included 615,661 mothers and 886,677 babies.

**EXPOSURES, OUTCOMES AND COVARIATES**

Pregnancy and pregnancy outcomes will be identified using the methods listed above. All women with at least one indication of pregnancy will be included in the pregnancy register. A Read code list of the existing codes for live birth is included as an appendix.

**DATA ANALYSIS**

All analyses will be conducted using Stata version 11.1. The following four stages of the project will be conducted.

Stage One

Linked HES data, including the information available in the delivery and birth HES data, but also standard HES data (including procedures and ICD codes), will be evaluated. The HES data will be seen, at this stage, as an additional data source for estimating the delivery date for the mother and the birth date for the baby. Additional information, where found, will be incorporated into the algorithm. The ease and efficiency of this process will be evaluated.

Stage Two

ONS mortality data will also be considered for potential dates of live births, although it is expected this will be utilised more in stage three of the study.

Stage Three

When the mother baby link is optimised for mothers and live births, the next stage will be to extend its remit to a full pregnancy register including pregnancies resulting in termination, miscarriage and stillbirth. This will require thorough and detailed searching of the data available in GPRD, HES and ONS. Methods to handle conflicting records will need to be established. The goal is a complete pregnancy history for each woman in GPRD, with details of pregnancy outcomes, estimated conception and delivery dates, and gestational age at birth.

Stage Four

A validation exercise will be undertaken once all information has been incorporated into the pregnancy register. This will involve searching the GPRD free text for evidence of pregnancies to establish the sensitivity and specificity of the mother baby link. Questionnaires will be sent to GPs to ask whether the match between the mother and baby is correct.

**USER GROUP INVOLVEMENT**

The key users of the Mother Baby Link will be GPRD users. As such, a number of academic groups with an interest in maternity data have reviewed this protocol and will be active collaborators on the study, especially in the areas of interpretation and plans for future work.

**LIMITATIONS**

Many babies do not have birth month in their GPRD records (and date of birth is not available) making matching between mother and baby more challenging. All matching between mothers and babies will be based on probabilistic methods, although the validation exercise will give an indication of sensitivity and specificity. Data from HES and ONS is limited by geography, practice consent and data coverage period. There are known data quality issues with HES delivery and birth data, which may prevent full utilisation of this data source.

**DISSEMINATION OF RESULTS**

The results of this study will be communicated to GPRD users and license holders. Findings will also be published for peer review in international scientific journals and conferences as appropriate.

It should be noted that all delivery dates will be estimated based upon probabilistic linkage, and GPRD will never release information as to whether a delivery date is a confirmed date of birth or not.

READ CODES FOR LIVE BIRTHS

| Read Code | Read Term |
| --- | --- |
| 6311 | Home birth |
| 6312 | GP unit birth |
| 6313 | Consultant unit birth |
| 6314 | Nursing home birth |
| 6315 | Ambulance birth |
| 6316 | Born before arrival |
| 6321 | 1st stage of labour length |
| 6322 | 2nd stage of labour length |
| 6323 | 3rd stage of labour length |
| 6331 | Single live birth |
| 6333 | Twins - both live born |
| 6334 | Twins - 1 still + 1 live born |
| 6335 | Twins - both still born |
| 6336 | Triplets - all live born |
| 6337 | Triplets -2 live+ 1 still born |
| 6341 | Baby male |
| 6342 | Baby female |
| 6343 | 2 male babies |
| 6344 | 2 female babies |
| 6345 | 1 male + 1 female baby |
| 6346 | 3 male babies |
| 6347 | 2 male + 1 female babies |
| 6348 | 1 male + 2 female babies |
| 6349 | 3 female babies |
| 6351 | Baby premature 36-38 weeks |
| 6352 | Baby v. premature 32-36 weeks |
| 6353 | Baby extremely prem.28-32 week |
| 6354 | Baby full term maturity |
| 6355 | Baby post-mature |
| 6356 | Baby premature 26-28 weeks |
| 6357 | Baby premature 24-26 weeks |
| 6358 | Baby premature 39 weeks |
| 6359 | Baby premature 38 weeks |
| 6361 | Baby BW = < 3% (under 2500g) |
| 6362 | Baby BW = 3% - 9% (2500-2849g) |
| 6363 | Baby BW = 10%-24% (2850-3149g) |
| 6364 | Baby BW = 25%-49% (3150-3449g) |
| 6365 | Baby BW = 50%-74% (3450-3749g) |
| 6366 | Baby BW = 75%-89% (3750-4049g) |
| 6367 | Baby BW = 90%-96% (4050-4399g) |
| 6368 | Baby BW = > 96% (over 4499g) |
| 6369 | Baby BW = 4400 - 4499g |
| 6371 | Birth HC = < 3rd centile |
| 6372 | Birth HC = 3rd-9th centile |
| 6373 | Birth HC = 10th-24th centile |
| 6374 | Birth HC = 25th-49th centile |
| 6375 | Birth HC = 50th-74th centile |
| 6376 | Birth HC = 75th-89th centile |
| 6377 | Birth HC = 90th-96th centile |
| 6378 | Birth HC = > 97th centile |
| 6381 | Birth length = < 3rd centile |
| 6382 | Birth length=3rd-9th centile |
| 6383 | Birth length=10th-24th centile |
| 6384 | Birth length=25th-49th centile |
| 6385 | Birth length=50th-74th centile |
| 6386 | Birth length=75th-89th centile |
| 6391 | Apgar at 1 minute = 0 |
| 6392 | Apgar at 1 minute = 1 |
| 6393 | Apgar at 1 minute = 2 |
| 6394 | Apgar at 1 minute = 3 |
| 6395 | Apgar at 1 minute = 4 |
| 6396 | Apgar at 1 minute = 5 |
| 6397 | Apgar at 1 minute = 6 |
| 6398 | Apgar at 1 minute = 7 |
| 6399 | Apgar at 1 minute = 8 |
| 6411 | Bottle fed at 10 days |
| 6412 | Breast fed at 10 days |
| 6413 | Breast + supp. fed at 10 days |
| 6421 | Bottle fed at 6 weeks |
| 6422 | Breast fed at 6 weeks |
| 6423 | Breast fed + supp. at 6 weeks |
| 6424 | On solids at 6 weeks |
| 6431 | Bottle fed at 3 months |
| 6432 | Breast fed at 3 months |
| 6433 | Breast + supp.fed at 3 months |
| 6434 | On solids at 3 months |
| 6441 | Bottle fed at 6 months |
| 6442 | Breast fed at 6 months |
| 6443 | Breast + supp. fed at 6 months |
| 6444 | On solids at 6 months |
| 6445 | On normal diet at 6 months |
| 1Y1..00 | Well baby |
| 62P..00 | Infant feeding method |
| 62P1.00 | Breast fed |
| 62P1.11 | Infant breast fed |
| 62P2.00 | Bottle fed |
| 62P2.11 | Infant bottle fed |
| 62P3.00 | Breast feeding with supplement |
| 62P4.00 | Breast changed to bottle feed |
| 62P5.00 | Breast feeding started |
| 62P6.00 | Breast feeding stopped |
| 62P7.00 | Bottle feeding started |
| 62P8.00 | Bottle feeding stopped |
| 62P9.00 | Infant weaned |
| 62PA.00 | Mother currently breast feeding |
| 62PB.00 | Bottle changed to breast |
| 62PC.00 | Breast feeding problem |
| 62PD.00 | Lactation established |
| 62PZ.00 | Infant feeding method NOS |
| 62Q..00 | Postnatal care provider |
| 62Q1.00 | P/N care from consultant |
| 62Q2.00 | P/N care from G.P. |
| 62Q3.00 | P/N - shared care |
| 62Q4.00 | No post natal care |
| 62Q5.00 | P/N care refused |
| 62Q6.00 | Postnatal care |
| 62QZ.00 | Post natal care NOS |
| 62R..00 | Postnatal visits |
| 62R1.00 | P/N - first day visit |
| 62R2.00 | P/N - second day visit |
| 62R3.00 | P/N - third day visit |
| 62R4.00 | P/N - fourth day visit |
| 62R5.00 | P/N - fifth day visit |
| 62R6.00 | P/N - sixth day visit |
| 62R7.00 | P/N - seventh day visit |
| 62R8.00 | P/N - eighth day visit |
| 62R9.00 | P/N - ninth day visit |
| 62RA.00 | P/N - tenth day visit |
| 62RB.00 | P/N care started at birth |
| 62RC.00 | P/N care <48hrs after birth |
| 62RD.00 | P/N care >48hrs after birth |
| 62RZ.00 | Postnatal visit NOS |
| 62S..00 | Maternal P/N 6 week exam. |
| 62S..11 | Postnatal exam. - maternal |
| 62S1.00 | Maternal P/N exam. not offered |
| 62S2.00 | Maternal P/N exam. offered |
| 62S3.00 | Maternal P/N exam. refused |
| 62S4.00 | Maternal P/N exam. defaulted |
| 62S5.00 | Maternal P/N exam. done |
| 62S6.00 | Postnatal examination minor problem found |
| 62S7.00 | Postnatal examination normal |
| 62SZ.00 | Maternal P/N 6 week exam. NOS |
| 62T..00 | Misc. postnatal data |
| 62T1.00 | Puerperal depression |
| 62TZ.00 | Misc. post natal data NOS |
| 62X4.00 | Length of gestation at birth |
| 63...00 | Birth details |
| 631..00 | Place of birth |
| 631..11 | Born - place delivered |
| 631Z.00 | Place of birth NOS |
| 632..00 | Length of labour |
| 632Z.00 | Length of labour NOS |
| 633..00 | Outcome of delivery |
| 633..11 | Livebirth |
| 633..13 | Triplet birth |
| 633..14 | Twin birth |
| 633a.00 | Birth of child |
| 633Z.00 | Outcome of delivery NOS |
| 634..00 | Sex of baby |
| 634..11 | Delivery - sex of baby |
| 634..12 | Female baby |
| 634..13 | Male baby |
| 634Z.00 | Sex of baby NOS |
| 635..00 | Maturity of baby |
| 635..11 | Full term baby |
| 635..12 | Postmature baby |
| 635..13 | Premature baby |
| 635A.00 | Baby premature 37 weeks |
| 635B.00 | Baby premature 36 weeks |
| 635Z.00 | Baby maturity NOS |
| 636..00 | Birthweight of baby |
| 636..11 | Birthweight |
| 636..12 | Weight - baby |
| 636A.00 | Baby BW = below 751gm |
| 636B.00 | Baby BW = 751g-1kg |
| 636C.00 | Baby BW = 1.0-1.5kg |
| 636D.00 | Baby BW = 1.5-2.0kg |
| 636E.00 | Baby BW = 2.0 - 2.5kg |
| 636F.00 | Baby BW = above 2.5kg |
| 636Z.00 | Birthweight of baby NOS |
| 637..00 | Birth head circumference |
| 637Z.00 | Birth head circumference NOS |
| 638..00 | Birth length |
| 638Z.00 | Birth length = > 97th centile |
| 639..00 | Apgar at 1 minute |
| 639A.00 | Apgar at 1 minute = 9 |
| 639B.00 | Apgar at 1 minute = 10 |
| 639Z.00 | Apgar at 1 minute NOS |
| 63A..00 | Apgar at 5 minutes |
| 63A1.00 | Apgar at 5 minutes = 0 |
| 63A2.00 | Apgar at 5 minutes = 1 |
| 63A3.00 | Apgar at 5 minutes = 2 |
| 63A4.00 | Apgar at 5 minutes = 3 |
| 63A5.00 | Apgar at 5 minutes = 4 |
| 63A6.00 | Apgar at 5 minutes = 5 |
| 63A7.00 | Apgar at 5 minutes = 6 |
| 63A8.00 | Apgar at 5 minutes = 7 |
| 63A9.00 | Apgar at 5 minutes = 8 |
| 63AA.00 | Apgar at 5 minutes = 9 |
| 63AB.00 | Apgar at 5 minutes = 10 |
| 63AZ.00 | Apgar at 5 minutes NOS |
| 63B..00 | Apgar at 10 minutes |
| 63B1.00 | Apgar at 10 minutes = 0 |
| 63B2.00 | Apgar at 10 minutes = 1 |
| 63B3.00 | Apgar at 10 minutes = 2 |
| 63B4.00 | Apgar at 10 minutes = 3 |
| 63B5.00 | Apgar at 10 minutes = 4 |
| 63B6.00 | Apgar at 10 minutes = 5 |
| 63B7.00 | Apgar at 10 minutes = 6 |
| 63B8.00 | Apgar at 10 minutes = 7 |
| 63B9.00 | Apgar at 10 minutes = 8 |
| 63BA.00 | Apgar at 10 minutes = 9 |
| 63BB.00 | Apgar at 10 minutes = 10 |
| 63BZ.00 | Apgar at 10 minutes NOS |
| 63C..00 | Baby misc. ""at-risk"" factors |
| 63C1.00 | Risk factor - been on SCBU |
| 63C1.11 | Risk factor - been on special care unit |
| 63C2.00 | Bonding problems |
| 63C3.00 | Cot death liability |
| 63C4.00 | Battered baby suspect - FH |
| 63C5.00 | Maternal tobacco abuse |
| 63C6.00 | Maternal drug abuse |
| 63C7.00 | Maternal alcohol abuse |
| 63C8.00 | Mother < 20 years old |
| 63C9.00 | Mother has a social worker |
| 63CA.00 | H.V.: mother not managing well |
| 63CB.00 | Risk of non-accidental injury |
| 63CC.00 | Difficult to establish feeding |
| 63CD.00 | High risk infant |
| 63CE.00 | One of twins |
| 63CE.11 | Fraternal twin |
| 63CE.12 | Heterozygous twin |
| 63CE.13 | Identical twin |
| 63CE.14 | Monozygous twin |
| 63CF.00 | One of triplets |
| 63CZ.00 | Baby ""at-risk"" factors NOS |
| 63D..00 | Placental details |
| 63D1.00 | Placental weight |
| 63D2.00 | Placenta normal O/E |
| 63D3.00 | Placenta diameter |
| 63D4.00 | Placental infarct |
| 63D5.00 | Placental abnormality |
| 63D6.00 | Placenta incomplete |
| 63DZ.00 | Placental details NOS |
| 63E..00 | Labour details |
| 63E1.00 | Spontaneous onset of labour |
| 63E2.00 | Normal birth |
| 63E3.00 | Normal labour |
| 63F..00 | Birth details not known |
| 63Z..00 | Birth details NOS |
| 63Z..11 | Apgar normal |
| 641..00 | Infant feeding - at 10 days |
| 641Z.00 | Infant feeding at 10 days NOS |
| 642..00 | Infant feeding at 6 weeks |
| 642Z.00 | Infant feeding at 6 weeks NOS |
| 643..00 | Infant feeding at 3 months |
| 643Z.00 | Infant feeding at 3 months NOS |
| 644..00 | Infant feeding at 6 months |
| 644Z.00 | Infant feeding at 6 months NOS |
| 64a..00 | Child 8 week exam |
| 64a1.00 | Child 8 week exam. not offered |
| 64a2.00 | Child 8 week exam. not wanted |
| 64a3.00 | Child 8 week exam.not attended |
| 64a4.00 | Child 8 week exam. normal |
| 64a5.00 | 8 week exam.abnormal -for obs. |
| 64a6.00 | 8 week exam.abnormal -referred |
| 64a7.00 | 8 week exam.abn.-on treatment |
| 64B..00 | Child exam. - birth |
| 64B1.00 | Child not examined at birth |
| 64B2.00 | Child birth exam. - normal |
| 64B2.11 | Baby normal at birth |
| 64B3.00 | Birth exam. abnormal -for obs. |
| 64B4.00 | Birth exam. abnormal -referred |
| 64B5.00 | Birth exam abn. - on treatment |
| 64BZ.00 | Child exam. - birth NOS |
| 64C..00 | Child exam. - 10 day |
| 64C1.00 | Child not examined at 10 days |
| 64C2.00 | Child 10 day exam. - normal |
| 64C3.00 | 10 day exam.abnormal -for obs. |
| 64C4.00 | 10 day exam. abnormal-referred |
| 64CZ.00 | Child 10 day exam. NOS |
| 64D..00 | Child 6 week exam. |
| 64D1.00 | Child 6 week exam. not offered |
| 64D2.00 | Child 6 week exam. not wanted |
| 64D3.00 | Child 6 week exam.not attended |
| 64D4.00 | Child 6 week exam. normal |
| 64D5.00 | 6 week exam.abnormal -for obs. |
| 64D6.00 | 6 week exam.abnormal -referred |
| 64D7.00 | 6 week exam.abn.-on treatment |
| 64DZ.00 | Child 6 week exam. NOS |
| 64e..00 | Infant feeding at birth |
| 64e0.00 | Bottle fed at birth |
| 64e1.00 | Breast fed at birth |
| 64f..00 | Infant feeding at 4 months |
| 64f0.00 | Breast fed at 4 months |
| 64T1.00 | Child 3 month examination |
| 64T2.00 | Child 6 month examination NEC |
| 64V..00 | Child 6 month examination |
| 64V4.00 | Child 6 month exam normal |
| 64V5.00 | Child 6/12 exam abnorm for obs |
| 64V6.00 | Child 6/12 exam abnorm: refer |
| 64VZ.00 | Child 6/12 exam NOS |
| 67C..00 | Postnatal support group |
| 7F...11 | Childbirth operations |
| 7F...13 | Puerperium operations |
| 7F1..00 | Induction and delivery operations |
| 7F1..11 | Labour operations |
| 7F10.00 | Surgical induction of labour |
| 7F10000 | Fore water rupture of amniotic membrane |
| 7F10100 | Hind water rupture of amniotic membrane |
| 7F10y00 | Other specified surgical induction of labour |
| 7F10z00 | Surgical induction of labour NOS |
| 7F10z11 | Artificial rupture of membranes |
| 7F10z12 | ARM |
| 7F11.00 | Other induction of labour |
| 7F11000 | Oxytocic induction of labour |
| 7F11100 | Induction of labour using prostaglandins |
| 7F11200 | Syntocinon induction of labour |
| 7F11300 | Medical induction of labour |
| 7F11y00 | Other specified other induction of labour |
| 7F11z00 | Other induction of labour NOS |
| 7F12.00 | Elective caesarean delivery |
| 7F12000 | Elective upper uterine segment caesarean delivery |
| 7F12100 | Elective lower uterine segment caesarean delivery |
| 7F12111 | Elective lower uterine segment caesarean section (LSCS) |
| 7F12y00 | Other specified elective caesarean delivery |
| 7F12z00 | Elective caesarean delivery NOS |
| 7F13.00 | Other caesarean delivery |
| 7F13000 | Upper uterine segment caesarean delivery NEC |
| 7F13100 | Lower uterine segment caesarean delivery NEC |
| 7F13111 | Lower uterine segment caesarean section (LSCS) NEC |
| 7F13200 | Extraperitoneal caesarean section |
| 7F13300 | Emergency caesarean section |
| 7F13y00 | Other specified other caesarean delivery |
| 7F13z00 | Other caesarean delivery NOS |
| 7F14.00 | Breech extraction delivery |
| 7F14100 | Forceps to aftercoming head (breech) |
| 7F14y00 | Other specified breech extraction delivery |
| 7F14z00 | Breech extraction delivery NOS |
| 7F15.00 | Other breech delivery |
| 7F15000 | Spontaneous breech delivery |
| 7F15100 | Assisted breech delivery |
| 7F15y00 | Other specified other breech delivery |
| 7F15z00 | Other breech delivery NOS |
| 7F16.00 | Forceps cephalic delivery |
| 7F16000 | High forceps cephalic delivery with rotation |
| 7F16100 | High forceps cephalic delivery NEC |
| 7F16200 | Mid forceps cephalic delivery with rotation |
| 7F16300 | Mid forceps cephalic delivery NEC |
| 7F16400 | Low forceps cephalic delivery |
| 7F16500 | Trial of forceps delivery |
| 7F16600 | Failed forceps delivery |
| 7F16700 | Barton forceps cephalic delivery with rotation |
| 7F16900 | Kielland forceps cephalic delivery with rotation |
| 7F16y00 | Other specified forceps cephalic delivery |
| 7F16z00 | Forceps cephalic delivery NOS |
| 7F17.00 | Vacuum delivery |
| 7F17.11 | Ventouse delivery |
| 7F17.12 | Ventouse extraction |
| 7F17100 | Low vacuum delivery |
| 7F17200 | Vacuum delivery before full dilation of cervix |
| 7F17300 | Trial of vacuum delivery |
| 7F17y00 | Other specified vacuum delivery |
| 7F17z00 | Vacuum delivery NOS |
| 7F18.00 | Cephalic vaginal deliv abnorm presentation head - no instrum |
| 7F18000 | Manip cephalic vaginal deliv abnorm pres head without instrm |
| 7F18100 | Nonmanip cephal vagin deliv abnorm pres head without instrum |
| 7F18y00 | Cephalic vagin deliv abnorm pres head without instrument OS |
| 7F18z00 | Cephalic vagin deliv abnorm pres head without instrument NOS |
| 7F19.00 | Normal delivery |
| 7F19000 | Manually assisted vaginal delivery |
| 7F19100 | Water birth delivery |
| 7F19y00 | Other specified normal delivery |
| 7F19z00 | Normal delivery NOS |
| 7F1A.00 | Other methods of delivery |
| 7F1A000 | Caesarean hysterectomy |
| 7F1A200 | Cleidotomy of fetus to facilitate delivery |
| 7F1A300 | Drainage of hydrocephalus of fetus to facilitate delivery |
| 7F1A400 | Trial of labour NEC |
| 7F1Ay00 | Other specified other method of delivery |
| 7F1Az00 | Other method of delivery NOS |
| 7F1B.00 | Other operations to facilitate delivery |
| 7F1B000 | Episiotomy to facilitate delivery |
| 7F1B100 | Symphysiotomy to facilitate delivery |
| 7F1B300 | Manual dilatation of cervix |
| 7F1B400 | Incision of cervix to facilitate delivery |
| 7F1Bz00 | Other operation to facilitate delivery NOS |
| 7F1y.00 | Other specified induction or delivery operations |
| 7F1z.00 | Induction and delivery operations NOS |
| 7F23.00 | Immediate repair of obstetric laceration |
| 7F23.11 | Immediate repair of obstetric tear |
| 7F23.12 | Immediate suture of obstetric laceration |
| 7F23000 | Immed repair obstetric laceration of uterus or cervix uteri |
| 7F23100 | Immed repair obstetric laceration perineum & anal sphincter |
| 7F23200 | Immed repair obstetric laceration vagina and floor of pelvis |
| 7F23300 | Immediate repair of minor obstetric laceration |
| 7F23400 | Repair of episiotomy |
| 7F23500 | Repair of ruptured uterus |
| 7F23y00 | Other specified immediate repair of obstetric laceration |
| 7F23z00 | Immediate repair of obstetric laceration NOS |
| 7F25.13 | Monitoring during labour |
| 7F25100 | Fetal heart monitoring in labour |
| 8HE7.00 | Discharged from hospital within 6 hours of delivery |
| 8Hl8.00 | Referral to breast feeding peer support service |
| L100100 | Threatened abortion - delivered |
| L10z100 | Early pregnancy haemorrhage NOS - delivered |
| L110100 | Placenta praevia without haemorrhage - delivered |
| L111100 | Placenta praevia with haemorrhage - delivered |
| L112100 | Placental abruption - delivered |
| L114100 | Antepartum haemorrhage with trauma - delivered |
| L115100 | Antepartum haemorrhage with uterine leiomyoma - delivered |
| L11y100 | Other antepartum haemorrhage - delivered |
| L11z100 | Antepartum haemorrhage NOS - delivered |
| L120100 | Benign essential hypertension in preg/childb/puerp - deliv |
| L120200 | Benign ess hypert in preg/childb/puerp - deliv with p/n comp |
| L120400 | Benign essential hypertension in preg/childb/puerp +p/n comp |
| L121100 | Renal hypertension in pregnancy/childbirth/puerp - delivered |
| L122100 | Other pre-existing hypertension in preg/childb/puerp - deliv |
| L123100 | Transient hypertension of pregnancy - delivered |
| L123200 | Transient hypertension of pregnancy - deliv with p/n comp |
| L123400 | Transient hypertension of pregnancy + postnatal complication |
| L124100 | Mild or unspecified pre-eclampsia - delivered |
| L124200 | Mild or unspecified pre-eclampsia - delivered with p/n comp |
| L124400 | Mild or unspecified pre-eclampsia with p/n complication |
| L125100 | Severe pre-eclampsia - delivered |
| L125200 | Severe pre-eclampsia - delivered with postnatal complication |
| L125400 | Severe pre-eclampsia with postnatal complication |
| L126100 | Eclampsia - delivered |
| L126200 | Eclampsia - delivered with postnatal complication |
| L126400 | Eclampsia with postnatal complication |
| L126600 | Eclampsia in labour |
| L127100 | Pre-eclampsia or eclampsia with hypertension - delivered |
| L127200 | Pre-eclampsia or eclampsia with hypertension - del+p/n comp |
| L12z100 | Unspecified hypertension in preg/childb/puerp - delivered |
| L12z200 | Unspecified hypertension in preg/childb/puerp -del +p/n comp |
| L130100 | Mild hyperemesis-delivered |
| L132100 | Late pregnancy vomiting - delivered |
| L13z100 | Unspecified pregnancy vomiting - delivered |
| L14..11 | Premature labour |
| L142.00 | Early onset of delivery |
| L142.11 | Premature delivery |
| L142000 | Early onset of delivery unspecified |
| L142100 | Early onset of delivery - delivered |
| L142z00 | Early onset of delivery NOS |
| L150100 | Post-term pregnancy - delivered |
| L162100 | Unspecified renal disease in pregnancy - delivered |
| L165200 | Asymptomatic bacteriuria in pregnancy - del with p/n comp |
| L166100 | Genitourinary tract infection in pregnancy - delivered |
| L166400 | Genitourinary tract infection in pregnancy with p/n comp |
| L166600 | Urinary tract infection following delivery |
| L167100 | Liver disorder in pregnancy - delivered |
| L168100 | Fatigue during pregnancy - delivered |
| L168400 | Fatigue during pregnancy with postnatal complication |
| L169200 | Herpes gestationis - delivered with postnatal complication |
| L169400 | Herpes gestationis with postnatal complication |
| L16A100 | Glycosuria during pregnancy - delivered |
| L16A200 | Glycosuria during pregnancy - delivered with p/n comp |
| L16y100 | Other pregnancy complication - delivered |
| L16y200 | Other pregnancy complication - delivered with postnatal comp |
| L170100 | Maternal syphilis during pregnancy - baby delivered |
| L175100 | Maternal rubella during pregnancy - baby delivered |
| L17y200 | Other mat.infect/parasit dis in puerperium - baby delivered |
| L180100 | Diabetes mellitus during pregnancy - baby delivered |
| L180400 | Diabetes mellitus in pueperium - baby previously delivered |
| L181100 | Thyroid dysfunction during pregnancy - baby delivered |
| L181500 | Postpartum thyroiditis |
| L182100 | Anaemia during pregnancy - baby delivered |
| L182200 | Anaemia in the puerperium - baby delivered |
| L182400 | Anaemia in the puerperium - baby previously delivered |
| L183100 | Drug dependence during pregnancy - baby delivered |
| L184400 | Mental disorder in puerperium - baby previously delivered |
| L185100 | Congenital cardiovasc dis in pregnancy - baby delivered |
| L185400 | Congenital cardiovasc dis in puerp - baby previously deliv |
| L188100 | Abnormal GTT during pregnancy - baby delivered |
| L188200 | Abnormal GTT in puerperium - baby delivered |
| L18z100 | Medical condition NOS during pregnancy - baby delivered |
| L20..00 | Normal delivery in a completely normal case |
| L20..11 | Spontaneous vaginal delivery |
| L200.00 | Normal delivery but ante- or post- natal conditions present |
| L20z.00 | Normal delivery in completely normal case NOS |
| L210100 | Twin pregnancy - delivered |
| L211100 | Triplet pregnancy - delivered |
| L213.00 | Multiple delivery |
| L213000 | Multiple delivery, all spontaneous |
| L213100 | Multiple delivery, all by forceps and vacuum extractor |
| L213200 | Multiple delivery, all by caesarean section |
| L21z100 | Multiple pregnancy NOS - delivered |
| L22..11 | Malpresentation of fetus |
| L220100 | Unstable lie - delivered |
| L221100 | Cephalic version NOS - delivered |
| L222.00 | Breech presentation |
| L222.11 | Assisted breech delivery |
| L222.12 | Breech delivery |
| L222.13 | Spontaneous breech delivery |
| L222000 | Breech presentation unspecified |
| L222100 | Breech presentation - delivered |
| L222200 | Breech presentation with antenatal problem |
| L222z00 | Breech presentation NOS |
| L223.00 | Oblique presentation |
| L224.00 | Transverse presentation |
| L224100 | Transverse lie - delivered |
| L224z11 | Shoulder presentation |
| L225.00 | Face presentation |
| L225000 | Face presentation unspecified |
| L225100 | Face presentation - delivered |
| L225z00 | Face presentation NOS |
| L226.00 | Brow presentation |
| L226100 | Brow presentation - delivered |
| L226200 | Brow presentation with antenatal problem |
| L227.00 | High head at term |
| L227z00 | High head at term NOS |
| L228.00 | Multiple pregnancy with malpresentation |
| L229.00 | Prolapsed arm presentation |
| L22y.11 | Compound presentation |
| L22z100 | Fetal malposition and malpresentation NOS - delivered |
| L23..00 | Cephalo-pelvic disproportion |
| L230.00 | Disproportion - major pelvic abnormality |
| L230000 | Disproportion - major pelvic abnormality unspecified |
| L230100 | Disproportion - major pelvic abnormality - delivered |
| L231.00 | Generally contracted pelvis |
| L231100 | Generally contracted pelvis - delivered |
| L231z00 | Generally contracted pelvis NOS |
| L232.00 | Inlet pelvic contraction |
| L233.00 | Outlet pelvic contraction |
| L233z00 | Outlet pelvic contraction NOS |
| L234.00 | Mixed feto-pelvic disproportion |
| L234100 | Mixed feto-pelvic disproportion - delivered |
| L235.00 | Large fetus causing disproportion |
| L235100 | Large fetus causing disproportion - delivered |
| L236.00 | Hydrocephalic disproportion |
| L236000 | Hydrocephalic disproportion unspecified |
| L236100 | Hydrocephalic disproportion - delivered |
| L236z00 | Hydrocephalic disproportion NOS |
| L237.00 | Other fetal abnormality causing disproportion |
| L237100 | Other fetal abnormality causing disproportion - delivered |
| L237200 | Other fetal abnormality causing disproportion with a/n prob |
| L23y.00 | Other disproportion |
| L23y100 | Other disproportion - delivered |
| L23z.00 | Disproportion NOS |
| L23z100 | Disproportion NOS - delivered |
| L23zz00 | Disproportion NOS |
| L240111 | Bicornuate uterus - baby delivered |
| L240211 | Bicornuate uterus - baby delivered + postpartum complication |
| L240400 | Cong abnorm uterus complic p/n care - baby previously deliv |
| L241100 | Tumour of uterine body - baby delivered |
| L241111 | Uterine fibroid - baby delivered |
| L241200 | Tumour of uterine body - baby delivered + p/n complication |
| L242100 | Uterine operation scar in pregnancy/childbirth/puerp - deliv |
| L244100 | Other uterine/pelvic floor abnormality - baby delivered |
| L244111 | Cystocele - baby delivered |
| L244112 | Rectocele - baby delivered |
| L244200 | Other uterine/pelvic floor abn - delivered+postpartum compl |
| L244211 | Cystocele - delivered with postpartum complication |
| L244212 | Rectocele - delivered with postpartum complication |
| L244400 | Other uterine/pelvic floor abn - baby delivered previously |
| L244411 | Cystocele complicating postpartum care - baby delivered prev |
| L244412 | Rectocele complicating postpartum care - baby delivered prev |
| L246100 | Other cervical abnormality - baby delivered |
| L246200 | Other cervical abnormality - baby delivered+postpartum compl |
| L246211 | Polyp of cervix - baby delivered+postpartum complication |
| L246411 | Polyp of cervix complicating p/n care - baby deliv prev |
| L247111 | Septate vagina - baby delivered |
| L24z400 | Pelvic soft tissue abnorm in preg/childb/puerp with p/n comp |
| L250100 | Fetus with central nervous system malformation - delivered |
| L251100 | Fetus with chromosomal abnormality - delivered |
| L252100 | Fetus with hereditary disease - delivered |
| L255100 | Fetus with drug damage - delivered |
| L25y100 | Fetus with other damage NEC - delivered |
| L25z100 | Fetus with damage NOS - delivered |
| L261100 | Rhesus isoimmunisation - delivered |
| L262100 | Other blood-group isoimmunisation - delivered |
| L263100 | Fetal distress - delivered |
| L263300 | Labour and delivery complicated by fetal heart rate anomaly |
| L263400 | Labour and delivery complic by meconium in amniotic fluid |
| L263500 | Lab+del comp fetal ht rate anom wth meconium in amnio fluid |
| L263600 | Labour+delivery complicatd by biochem evidence/fetal stress |
| L264100 | Intrauterine death - delivered |
| L265100 | Small-for-dates - delivered |
| L266100 | Large-for-dates - delivered |
| L270100 | Polyhydramnios - delivered |
| L280100 | Oligohydramnios - delivered |
| L281100 | Premature rupture of membranes - delivered |
| L281300 | Prem rupture of membranes onset of labour within 24 hours |
| L281400 | Premature rupture of membranes, labour delayed by therapy |
| L281500 | Prem rupture of membranes onset of labour after 24 hours |
| L282100 | Prolonged spont/unspec rupture of membranes - delivered |
| L284100 | Amniotic cavity infection - delivered |
| L28y100 | Other problem of amniotic cavity and membranes - delivered |
| L29..00 | Other problems affecting labour |
| L290.00 | Failed mechanical induction |
| L290.11 | Failed mechanical induction of labour |
| L291.00 | Failed medical or unspecified induction |
| L291.11 | Failed medical induction of labour |
| L291100 | Failed medical or unspecified induction - delivered |
| L291z00 | Failed medical or unspecified induction NOS |
| L292.00 | Maternal pyrexia during labour, unspecified |
| L292100 | Unspecified maternal pyrexia during labour - delivered |
| L293100 | Septicaemia during labour - delivered |
| L294.00 | Grand multiparity |
| L294000 | Grand multiparity unspecified |
| L294100 | Grand multiparity - delivered |
| L294z00 | Grand multiparity NOS |
| L295.00 | Elderly primigravida |
| L296.00 | Vaginal delivery following previous caesarean section |
| L29z.00 | Problems affecting labour NOS |
| L29zz00 | Problems affecting labour NOS |
| L3...00 | Complications occurring during labour and delivery |
| L30..00 | Obstructed labour |
| L300.00 | Obstructed labour due to fetal malposition |
| L300100 | Obstructed labour due to fetal malposition - delivered |
| L300300 | Obstructed labour due to breech presentation |
| L300400 | Obstructed labour due to face presentation |
| L300700 | Obstructed labour due to compound presentation |
| L301.00 | Obstructed labour caused by bony pelvis |
| L301400 | Obstructed labour due to generally contracted pelvis |
| L301500 | Obstructed labour due to pelvic inlet contraction |
| L302.00 | Obstructed labour caused by pelvic soft tissues |
| L302000 | Obstructed labour caused by pelvic soft tissues unspecified |
| L303.00 | Deep transverse arrest (DTA) |
| L303000 | Deep transverse arrest unspecified |
| L303100 | Deep transverse arrest - delivered |
| L303z00 | Deep transverse arrest NOS |
| L304.00 | Persistent occipitoposterior or occipitoanterior position |
| L304100 | Persistent occipitopost/occipitoant position - delivered |
| L305.00 | Shoulder dystocia |
| L305.11 | Impacted shoulders |
| L305000 | Shoulder dystocia unspecified |
| L305100 | Shoulder dystocia - delivered |
| L305200 | Shoulder dystocia with antenatal problem |
| L305z00 | Shoulder dystocia NOS |
| L306.00 | Locked twins |
| L306100 | Locked twins - delivered |
| L307.00 | Failed trial of labour unspecified |
| L307200 | Other failed trial of labour with antenatal problem |
| L307z00 | Failed trial of labour NOS |
| L308.00 | Failed forceps unspecified |
| L308100 | Other failed forceps - delivered |
| L308z00 | Failed forceps NOS |
| L309.00 | Failed ventouse extraction unspecified |
| L309000 | Other failed ventouse extraction, unspecified |
| L309100 | Other failed ventouse extraction - delivered |
| L309z00 | Failed ventouse extraction NOS |
| L30z.00 | Obstructed labour NOS |
| L30z000 | Obstructed labour NOS, unspecified |
| L30z100 | Obstructed labour NOS - delivered |
| L30zz00 | Obstructed labour NOS |
| L30zz11 | Dystocia NOS |
| L31..00 | Abnormal forces of labour |
| L310.00 | Primary uterine inertia |
| L311.00 | Secondary uterine inertia |
| L311200 | Secondary uterine inertia with antenatal problem |
| L312.00 | Other uterine inertia |
| L312.11 | Atony of uterus |
| L312.12 | Poor contractions |
| L312100 | Other uterine inertia - delivered |
| L312200 | Other uterine inertia with antenatal problem |
| L312z00 | Other uterine inertia NOS |
| L313.00 | Precipitate labour |
| L313100 | Precipitate labour - delivered |
| L313z00 | Precipitate labour NOS |
| L314.12 | Contraction ring (dystocia) |
| L314.13 | Hourglass uterine contraction |
| L314.14 | Incoordinate uterine action |
| L314.15 | Uterine dystocia NOS |
| L314.16 | Uterine or cervical spasm |
| L314000 | Hypertonic uterine inertia unspecified |
| L31z.00 | Abnormality of forces of labour NOS |
| L32..00 | Long labour |
| L320.00 | Prolonged first stage |
| L320100 | Prolonged first stage - delivered |
| L320z00 | Prolonged first stage NOS |
| L321.00 | Prolonged labour unspecified |
| L321000 | Unspecified prolonged labour, unspecified |
| L321100 | Unspecified prolonged labour - delivered |
| L322.00 | Prolonged second stage |
| L322000 | Prolonged second stage unspecified |
| L322100 | Prolonged second stage - delivered |
| L322z00 | Prolonged second stage NOS |
| L323.00 | Delayed delivery of second twin, triplet etc |
| L323000 | Delayed delivery second twin unspecified |
| L323100 | Delayed delivery second twin - delivered |
| L323200 | Delayed delivery second twin with antenatal problem |
| L32z.00 | Prolonged labour NOS |
| L33..00 | Umbilical cord complications |
| L330.00 | Prolapse of cord |
| L330.11 | Presentation of cord |
| L330000 | Prolapse of cord unspecified |
| L330100 | Prolapse of cord - delivered |
| L330z00 | Prolapse of cord NOS |
| L331.00 | Cord tight round neck |
| L331000 | Cord tight round neck unspecified |
| L331100 | Cord tight round neck - delivered |
| L331z00 | Cord tight round neck NOS |
| L332.00 | Cord tangled or knotted with compression |
| L332.11 | Knot in cord |
| L333.00 | Other cord entanglement |
| L333100 | Other cord entanglement - delivered |
| L333z00 | Other cord entanglement NOS |
| L334.00 | Short cord |
| L335.00 | Vasa praevia |
| L335.11 | Velamentous insertion of cord |
| L336.00 | Vascular lesions of cord |
| L336.11 | Bruising of cord |
| L336000 | Vascular lesions of cord unspecified |
| L336z00 | Vascular lesions of cord NOS |
| L33y.00 | Other umbilical cord complications |
| L33y200 | Other umbilical cord complications with antenatal problem |
| L33yz00 | Other umbilical cord complications NOS |
| L33z.00 | Umbilical cord complications NOS |
| L33z000 | Umbilical cord complications NOS, unspecified |
| L33z100 | Umbilical cord complications NOS - delivered |
| L33z200 | Umbilical cord complications NOS with antenatal problem |
| L33zz00 | Umbilical cord complications NOS |
| L34..00 | Trauma to perineum and vulva during delivery |
| L34..11 | Perineal tear |
| L34..12 | Vulval delivery trauma |
| L340.00 | First degree perineal tear during delivery |
| L340.11 | Fourchette tear |
| L340.12 | Hymen tear |
| L340.13 | Labial tear |
| L340.14 | Vaginal tear |
| L340.15 | Vulval tear |
| L340000 | First degree perineal tear during delivery, unspecified |
| L340100 | First degree perineal tear during delivery - delivered |
| L340200 | First degree perineal tear during delivery with p/n problem |
| L340300 | Labial tear during delivery |
| L340400 | Fourchette tear during delivery |
| L340500 | Vulval tear during delivery |
| L340600 | Vaginal tear during delivery |
| L340z00 | First degree perineal tear during delivery NOS |
| L341.00 | Second degree perineal tear during delivery |
| L341.11 | Pelvic floor tear |
| L341.12 | Perineal muscle tear |
| L341.13 | Vaginal muscle tear |
| L341000 | Second degree perineal tear during delivery, unspecified |
| L341100 | Second degree perineal tear during delivery - delivered |
| L341200 | Second degree perineal tear during delivery with p/n prob |
| L341z00 | Second degree perineal tear during delivery NOS |
| L342.00 | Third degree perineal tear during delivery |
| L342.11 | Anal sphincter tear |
| L342000 | Third degree perineal tear during delivery, unspecified |
| L342100 | Third degree perineal tear during delivery - delivered |
| L342200 | Third degree perineal tear during delivery with p/n problem |
| L342z00 | Third degree perineal tear during delivery NOS |
| L343.00 | Fourth degree perineal tear during delivery |
| L343.11 | Mucosal tear of anus or rectum |
| L343000 | Fourth degree perineal tear during delivery, unspecified |
| L343100 | Fourth degree perineal tear during delivery - delivered |
| L343200 | Fourth degree perineal tear during delivery with p/n problem |
| L343z00 | Fourth degree perineal tear during delivery NOS |
| L344.00 | Unspecified perineal laceration during delivery |
| L344000 | Unspecified perineal laceration during delivery, unspecified |
| L344100 | Unspecified perineal laceration during delivery - delivered |
| L344200 | Unspecified perineal laceration during delivery + p/n prob |
| L344z00 | Unspecified perineal laceration during delivery NOS |
| L345.00 | Vulval and perineal haematoma during delivery |
| L345.11 | Perineal haematoma |
| L345.12 | Vulval and perineal haematoma during delivery |
| L345000 | Vulval and perineal haematoma during delivery, unspecified |
| L345100 | Vulval and perineal haematoma during delivery - delivered |
| L345200 | Vulval and perineal haematoma during delivery + p/n problem |
| L345z00 | Vulval and perineal haematoma during delivery NOS |
| L34y.00 | Other vulval and perineal trauma during delivery |
| L34y000 | Other vulval/perineal trauma during delivery, unspecified |
| L34y100 | Other vulval/perineal trauma during delivery- delivered |
| L34y200 | Other vulval/perineal trauma during delivery + p/n problem |
| L34yz00 | Other vulval/perineal trauma during delivery NOS |
| L34z.00 | Vulval/perineal trauma during delivery NOS |
| L34z000 | Vulval/perineal trauma during delivery NOS unspec |
| L34z100 | Vulval/perineal trauma during delivery NOS - delivered |
| L34zz00 | Vulval/perineal trauma during delivery NOS |
| L35..00 | Other obstetric trauma |
| L350.00 | Ruptured uterus before labour |
| L350000 | Rupture of uterus before labour unspecified |
| L350200 | Rupture of uterus before labour with antenatal problem |
| L350z00 | Rupture of uterus before labour NOS |
| L351.00 | Rupture of uterus during and after labour |
| L351000 | Rupture of uterus during and after labour unspecified |
| L351100 | Rupture of uterus during and after labour - delivered |
| L351300 | Rupture of uterus during/after labour with postnatal problem |
| L351z00 | Rupture of uterus during and after labour NOS |
| L352.00 | Obstetric inversion of uterus |
| L352.11 | Inversion of uterus - obstetric |
| L352200 | Obstetric inversion of uterus with postnatal problem |
| L352z00 | Obstetric inversion of uterus NOS |
| L353.00 | Obstetric laceration of cervix |
| L353.11 | Laceration of cervix - obstetric |
| L353.12 | Tear of cervix - obstetric |
| L353000 | Obstetric laceration of cervix unspecified |
| L353z00 | Obstetric laceration of cervix NOS |
| L354.00 | Obstetric high vaginal laceration |
| L354.11 | High vaginal laceration - obstetric |
| L354.12 | High vaginal tear - obstetric |
| L354000 | Obstetric high vaginal laceration unspecified |
| L354100 | Obstetric high vaginal laceration - delivered |
| L355.00 | Other obstetric pelvic organ damage |
| L355.11 | Bladder injury - obstetric |
| L355.12 | Urethra injury - obstetric |
| L355100 | Other obstetric pelvic organ damage - delivered |
| L356.00 | Obstetric trauma damaging pelvic joints and ligaments |
| L356.11 | Obstetric pelvic joint damage |
| L356.12 | Obstetric pelvic ligament damage |
| L356.13 | Pubic symphysis separation |
| L356.14 | Symphysis pubis separation |
| L356000 | Obstetric damage to pelvic joints and ligaments unspecified |
| L356200 | Obstetric damage to pelvic joints and ligaments + p/n prob |
| L356z00 | Obstetric damage to pelvic joints and ligaments NOS |
| L357.00 | Obstetric trauma causing pelvic haematoma |
| L357000 | Obstetric pelvic haematoma unspecified |
| L357100 | Obstetric pelvic haematoma - delivered |
| L35y.00 | Other obstetric trauma OS |
| L35y100 | Other obstetric trauma - delivered |
| L35y200 | Other obstetric trauma - delivered with postnatal problem |
| L35z.00 | Obstetric trauma NOS |
| L35z000 | Obstetric trauma NOS, unspecified |
| L35z100 | Obstetric trauma NOS - delivered |
| L35z400 | Obstetric trauma NOS with postnatal problem |
| L35zz00 | Obstetric trauma NOS |
| L36..00 | Postpartum haemorrhage (PPH) |
| L36..11 | Bleeding postpartum |
| L360.00 | Third-stage postpartum haemorrhage |
| L360.11 | Retained placenta NOS |
| L360000 | Third-stage postpartum haemorrhage unspecified |
| L360100 | Third-stage postpartum haemorrhage - deliv with p/n problem |
| L360200 | Third-stage postpartum haemorrhage with postnatal problem |
| L360z00 | Third-stage postpartum haemorrhage NOS |
| L361.00 | Other immediate postpartum haemorrhage |
| L361z00 | Other immediate postpartum haemorrhage NOS |
| L362.00 | Secondary and delayed postpartum haemorrhage |
| L362000 | Secondary postpartum haemorrhage unspecified |
| L362200 | Secondary postpartum haemorrhage with postnatal problem |
| L362z00 | Secondary and delayed postpartum haemorrhage NOS |
| L363.00 | Postpartum coagulation defects |
| L363z00 | Postpartum coagulation defects NOS |
| L36z.00 | Postpartum haemorrhage NOS |
| L37..00 | Retained placenta or membranes with no haemorrhage |
| L37..11 | Retained membrane without haemorrhage |
| L370.00 | Retained placenta with no haemorrhage |
| L370.11 | Placenta accreta without haemorrhage |
| L370000 | Retained placenta with no haemorrhage unspecified |
| L370z00 | Retained placenta with no haemorrhage NOS |
| L370z11 | Retained placenta without haemorrhage |
| L371.00 | Retained portion of placenta or membranes - no haemorrhage |
| L371000 | Retained products with no haemorrhage unspecified |
| L371100 | Retained products with no haemorrhage - deliv with p/n prob |
| L371200 | Retained products with no haemorrhage with postnatal problem |
| L371z00 | Retained products with no haemorrhage NOS |
| L37z.00 | Retained placenta or membranes with no haemorrhage NOS |
| L38..00 | Complications of anaesthesia during labour and delivery |
| L380.00 | Obstetric anaesthesia with pulmonary complications |
| L380.11 | Mendelson's syndrome |
| L382000 | Obstetric anaesthesia with CNS complications unspecified |
| L383.00 | Obstetric toxic reaction to local anaesthesia |
| L384.00 | Obstetric spinal and epidural anaesthesia-induced headache |
| L384100 | Spinal/epidural anaesth-induced headache during puerp |
| L385.00 | Failed or difficult intubation during pregnancy |
| L386.00 | Toxic reaction to local anaesthesia during labour and deliv |
| L387.00 | Spinal/epidural anesth-induced headache dur labour/delivery |
| L388.00 | Cardiac comps of anaesthesia during labour and delivery |
| L389.00 | CNS comps of anaesthesia during labour and delivery |
| L38A.00 | Failed or difficult intubation during labour and delivery |
| L38B.00 | Failed or difficult intubation during the puerperium |
| L39..00 | Other complications of labour and delivery NEC |
| L390.00 | Maternal distress |
| L391.00 | Obstetric shock |
| L391000 | Obstetric shock unspecified |
| L392.00 | Maternal hypotension syndrome |
| L392000 | Maternal hypotension syndrome unspecified |
| L392z00 | Maternal hypotension syndrome NOS |
| L393.00 | Acute renal failure following labour and delivery |
| L393000 | Post-delivery acute renal failure unspecified |
| L393100 | Post-delivery acute renal failure - delivered with p/n prob |
| L393200 | Post-delivery acute renal failure with postnatal problem |
| L394.00 | Other complications of obstetric procedures |
| L394100 | Other complications of obstetric procedures - delivered |
| L394200 | Other complications of obstetric procedures - del +p/n prob |
| L394500 | Infection of obstetric surgical wound |
| L394600 | Haematoma of obstetric wound |
| L394z00 | Other complications of obstetric procedures NOS |
| L395.00 | Forceps delivery |
| L395.11 | Keilland's forceps delivery |
| L395.12 | Neville - Barnes forceps delivery |
| L395.13 | Simpson's forceps delivery |
| L395000 | Forceps delivery unspecified |
| L395100 | Forceps delivery - delivered |
| L395200 | Low forceps delivery |
| L395300 | Mid-cavity forceps delivery |
| L395400 | Delivery by combination of forceps and vacuum extractor |
| L395500 | Mid-cavity forceps with rotation |
| L395z00 | Forceps delivery NOS |
| L396.00 | Vacuum extractor delivery |
| L396.11 | Ventouse delivery |
| L396000 | Vacuum extractor delivery unspecified |
| L396100 | Vacuum extractor delivery - delivered |
| L396z00 | Vacuum extractor delivery NOS |
| L397.00 | Breech extraction |
| L397000 | Breech extraction unspecified |
| L397100 | Breech extraction - delivered |
| L397z00 | Breech extraction NOS |
| L398.00 | Caesarean delivery |
| L398000 | Caesarean delivery unspecified |
| L398100 | Caesarean delivery - delivered |
| L398200 | Caesarean section - pregnancy at term |
| L398300 | Delivery by elective caesarean section |
| L398400 | Delivery by emergency caesarean section |
| L398500 | Delivery by caesarean hysterectomy |
| L398600 | Caesarean delivery following previous Caesarean delivery |
| L398z00 | Caesarean delivery NOS |
| L39A.00 | Death obst cse occur more 42 day less than one yr aft deliv |
| L39B.00 | Death from sequelae of direct obstetric causes |
| L39X.00 | Obstetric death of unspecified cause |
| L39y.00 | Other complications of labour and delivery |
| L39y100 | Other complications of labour and delivery - delivered |
| L39y400 | Other complications of labour and delivery with p/n problem |
| L39y411 | Postnatal vaginal discomfort |
| L39y412 | Vaginal discomfort postnatal |
| L39y500 | Maternal exhaustion |
| L39yz00 | Other complications of labour and delivery NOS |
| L39z.00 | Complications of labour and delivery NOS |
| L39z100 | Complications of labour and delivery NOS - delivered |
| L39z400 | Complications of labour and delivery NOS with p/n problem |
| L39zz00 | Complications of labour and delivery NOS |
| L3A..00 | Intrapartum haemorrhage with coagulation defect |
| L3X..00 | Intrapartum haemorrhage, unspecified |
| L3y..00 | Other specified complications of labour or delivery |
| L3z..00 | Complications of labour and delivery NOS |
| L4...00 | Complications of the puerperium |
| L40..00 | Major puerperal infection |
| L40..11 | Sepsis - puerperal |
| L400.00 | Puerperal endometritis |
| L400000 | Puerperal endometritis unspecified |
| L400100 | Puerperal endometritis - delivered with postnatal comp |
| L400200 | Puerperal endometritis with postnatal complication |
| L400z00 | Puerperal endometritis NOS |
| L401.00 | Puerperal salpingitis |
| L401200 | Puerperal salpingitis with postnatal complication |
| L401z00 | Puerperal salpingitis NOS |
| L402.00 | Puerperal peritonitis |
| L402000 | Puerperal peritonitis unspecified |
| L403.00 | Puerperal septicaemia |
| L403000 | Puerperal septicaemia unspecified |
| L40z.00 | Major puerperal infection NOS |
| L40z000 | Major puerperal infection NOS, unspecified |
| L40zz00 | Major puerperal infection NOS |
| L410400 | Varicose veins of legs in pregnancy/puerperium + p/n comp |
| L410600 | Varicose veins of legs in the puerperium |
| L411600 | Genital varices in the puerperium |
| L411612 | Vaginal varices in the puerperium |
| L412200 | Superficial thrombophleb in preg/puerperium - del + p/n comp |
| L412211 | Phlebitis - postpartum |
| L412212 | Puerperal phlebitis |
| L412400 | Superficial thrombophlebitis in preg/puerperium + p/n comp |
| L412600 | Superficial thrombophlebitis in the puerperium |
| L412611 | Thombophlebitis of legs in the puerperium |
| L413100 | Antenatal deep vein thrombosis - delivered |
| L414.00 | Postnatal deep vein thrombosis |
| L414.11 | DVT - deep venous thrombosis, postnatal |
| L414000 | Postnatal deep vein thrombosis unspecified |
| L414200 | Postnatal deep vein thrombosis with postnatal complication |
| L414z00 | Postnatal deep vein thrombosis NOS |
| L415600 | Other phlebitis in the puerperium |
| L416100 | Haemorrhoids in pregnancy and the puerperium - delivered |
| L416200 | Haemorrhoids in pregnancy and puerperium - deliv + p/n comp |
| L416500 | Haemorrhoids in the puerperium |
| L417100 | Cerebral venous thrombosis in the puerperium |
| L41z600 | Venous complication in the puerperium, unspecified |
| L41z611 | Puerperal phlebitis NOS |
| L41z613 | Puerperal thrombosis NOS |
| L42..00 | Puerperal pyrexia of unknown origin |
| L420.00 | Puerperal pyrexia of unknown origin |
| L420000 | Puerperal pyrexia of unknown origin unspecified |
| L420z00 | Puerperal pyrexia NOS |
| L42z.00 | Puerperal pyrexia NOS |
| L431100 | Amniotic fluid pulmonary embolism - delivered |
| L431200 | Amniotic fluid pulm embolism - delivered + p/n complication |
| L43z100 | Obstetric pulmonary embolism NOS - delivered |
| L43z400 | Obstetric pulmonary embolism NOS with postnatal complication |
| L44..00 | Other complications of the puerperium NEC |
| L440.00 | Cerebrovascular disorders in the puerperium |
| L440.11 | CVA - cerebrovascular accident in the puerperium |
| L440.12 | Stroke in the puerperium |
| L440000 | Puerperal cerebrovascular disorder unspecified |
| L440100 | Puerperal cerebrovascular disorder - delivered |
| L440300 | Puerperal cerebrovascular disorder with antenatal comp |
| L441.00 | Caesarean wound disruption |
| L441000 | Caesarean wound disruption unspecified |
| L441200 | Caesarean wound disruption with postnatal complication |
| L441z00 | Caesarean wound disruption NOS |
| L442.00 | Obstetric perineal wound disruption |
| L442.11 | Breakdown of perineum |
| L442.12 | Episiotomy breakdown |
| L442000 | Obstetric perineal wound disruption unspecified |
| L442100 | Obstetric perineal wound disruption - deliv + p/n comp |
| L442200 | Obstetric perineal wound disruption with p/n complication |
| L442z00 | Obstetric perineal wound disruption NOS |
| L443.00 | Other complication of obstetric surgical wound |
| L443.11 | Haematoma - perineal wound |
| L443.12 | Infection - perineal wound |
| L443000 | Other complication of obstetric surgical wound unspecified |
| L443200 | Other complication obstetric surgical wound with p/n comp |
| L443z00 | Other complication of obstetric surgical wound NOS |
| L444.00 | Placental polyp |
| L44y.00 | Other complications of the puerperium |
| L44y.11 | Subinvolution of uterus in the puerperium |
| L44y000 | Other complications of the puerperium unspecified |
| L44y100 | Other complications of the puerperium - delivered + p/n comp |
| L44yz00 | Other complications of the puerperium NOS |
| L44yz11 | Blood dyscrasia puerperal |
| L44z.00 | Complications of the puerperium NOS |
| L44z000 | Complications of the puerperium NOS, unspecified |
| L44zz00 | Complications of the puerperium NOS |
| L45..00 | Obstetric breast infections |
| L450.00 | Obstetric nipple infection |
| L450.11 | Abscess of nipple - obstetric |
| L450.12 | Nipple infection - obstetric |
| L450000 | Obstetric nipple infection unspecified |
| L450100 | Obstetric nipple infection - delivered |
| L450400 | Obstetric nipple infection with postnatal complication |
| L450z00 | Obstetric nipple infection NOS |
| L451.00 | Obstetric breast abscess |
| L451.11 | Purulent mastitis - obstetric |
| L451000 | Obstetric breast abscess unspecified |
| L451100 | Obstetric breast abscess - delivered |
| L451400 | Obstetric breast abscess with postnatal complication |
| L451z00 | Obstetric breast abscess NOS |
| L452.00 | Obstetric nonpurulent mastitis |
| L452.11 | Lymphangitis of breast - obstetric |
| L452000 | Obstetric nonpurulent mastitis unspecified |
| L452100 | Obstetric nonpurulent mastitis - delivered |
| L452200 | Obstetric nonpurulent mastitis - deliv with p/n complication |
| L452300 | Obstetric nonpurulent mastitis with antenatal complication |
| L452400 | Obstetric nonpurulent mastitis with postnatal complication |
| L452z00 | Obstetric nonpurulent mastitis NOS |
| L45y.00 | Other obstetric breast infections |
| L45y000 | Other obstetric breast infection unspecified |
| L45y100 | Other obstetric breast infection - delivered |
| L45z.00 | Obstetric breast infection NOS |
| L45z000 | Obstetric breast infection NOS, unspecified |
| L45z100 | Obstetric breast infection NOS - delivered |
| L45z400 | Obstetric breast infection NOS with postnatal complication |
| L45zz00 | Obstetric breast infection NOS |
| L46..00 | Obstetric breast and lactation disorders NOS |
| L46..11 | Lactation problems |
| L460.00 | Retracted nipple in pregnancy, the puerperium or lactation |
| L460000 | Retracted nipple in pregnancy/puerperium/lactation unspec |
| L460300 | Retracted nipple in pregnancy/puerperium/lact with a/n comp |
| L460z00 | Retracted nipple in pregnancy/puerperium/lactation NOS |
| L461.00 | Cracked nipple in pregnancy, the puerperium or lactation |
| L461.11 | Fissure of nipple |
| L461000 | Cracked nipple in pregnancy/puerperium/lactation unspecified |
| L461100 | Cracked nipple in pregnancy/puerperium/lactation - delivered |
| L461200 | Cracked nipple in pregnancy/puerp/lact - deliv + p/n comp |
| L461400 | Cracked nipple in pregnancy/puerperium/lactation + p/n comp |
| L461z00 | Cracked nipple in pregnancy, the puerperium or lactation NOS |
| L462.00 | Breast engorgement in pregnancy, the puerperium or lactation |
| L462000 | Breast engorgement in pregnancy/puerperium/lactation unspec |
| L462100 | Breast engorgement in pregnancy/puerperium/lactation - deliv |
| L462200 | Breast engorgement in pregnancy/puerp/lact - del + p/n comp |
| L462300 | Breast engorgement in pregnancy/puerperium/lact + a/n comp |
| L462400 | Breast engorgement in pregnancy/puerperium/lact + p/n comp |
| L462z00 | Breast engorgement in pregnancy/puerperium/lactation NOS |
| L462z11 | Breast engorgement |
| L463.00 | Other breast disorder in pregnancy/puerperium/lactation |
| L463000 | Other breast disorder in pregnancy/puerperium/lact unspec |
| L463300 | Other breast disorder in pregnancy/puerperium/lact +a/n comp |
| L463400 | Other breast disorder in pregnancy/puerperium/lact +p/n comp |
| L463500 | Pain on breast feeding |
| L463z00 | Other breast disorder in pregnancy/puerperium/lactation NOS |
| L464.00 | Failure of lactation |
| L464.11 | Agalactia |
| L465.00 | Suppressed lactation |
| L465000 | Suppressed lactation unspecified |
| L465z00 | Suppressed lactation NOS |
| L466.00 | Galactorrhoea in pregnancy and the puerperium |
| L466000 | Galactorrhoea in pregnancy and the puerperium unspecified |
| L466100 | Galactorrhoea in pregnancy and the puerperium - delivered |
| L466300 | Galactorrhoea in pregnancy/puerperium with a/n complication |
| L466z00 | Galactorrhoea in pregnancy and the puerperium NOS |
| L467.00 | Hypogalactia |
| L46y.00 | Other disorders of lactation |
| L46y.11 | Galactocele - obstetric |
| L46y000 | Other disorder of lactation unspecified |
| L46y100 | Other disorder of lactation - delivered |
| L46y400 | Other disorder of lactation with postnatal complication |
| L46yz00 | Other disorder of lactation NOS |
| L46z.00 | Disorders of lactation NOS |
| L46z000 | Disorder of lactation NOS, unspecified |
| L46zz00 | Disorder of lactation NOS |
| L4y..00 | Other specified complications of the puerperium |
| L4z..00 | Complications of the puerperium NOS |
| L50..00 | Maternal care for compound presentation |
| Ly0..00 | Spontaneous vertex delivery |
| Ly1..00 | Spontaneous breech delivery |
| Lyu4000 | [X]Other failed induction of labour |
| Lyu4100 | [X]Other uterine inertia |
| Lyu4500 | [X]Obstructed labour due to other abnormalities of fetus |
| Lyu4800 | [X]Labour+delivery complicat/oth evidence of fetal distress |
| Lyu4900 | [X]Labour+delivery complicated by other cord entanglement |
| Lyu4D00 | [X]Other immediate postpartum haemorrhage |
| Lyu5.00 | [X]Delivery |
| Lyu5000 | [X]Other single spontaneous delivery |
| Lyu5100 | [X]Other and unspecified forceps delivery |
| Lyu5200 | [X]Other single delivery by caesarean section |
| Lyu5400 | [X]Other manipulation-assisted delivery |
| Lyu5500 | [X]Other specified assisted single delivery |
| Lyu5700 | [X]Assisted single delivery, unspecified |
| Lyu6000 | [X]Other infection of genital tract following delivery |
| Lyu6100 | [X]Other genitourinary tract infections following delivery |
| Lyu6200 | [X]Other specified puerperal infection |
| Lyu6300 | [X]Other venous complications in the puerperium |
| Lyu6700 | [X]Other specified puerperal complications |
| Lyu6A00 | [X]Infection of caesarean section wound following delivery |
| Lyu6B00 | [X]Vaginitis following delivery |
| Lyu6C00 | [X]Cervicitis following delivery |
| Q021300 | Fetus/neonate affected by placental damage-caesarean section |
| Q021400 | Fetus/neonate affect by placental damage-surgical induction |
| Q03..00 | Fetus or neonate affected by complication of labour/delivery |
| Q030.00 | Fetus or neonate affected by breech delivery and extraction |
| Q030.11 | Fetus affected by breech delivery |
| Q031.00 | Fetus/neonate affected by malposition/disproportion-delivery |
| Q031000 | Fetus/neonate affected by mat bony pelvis abn in labour/del |
| Q031200 | Fetus/neonate affect persistent occip-posterior - labour/del |
| Q031300 | Fetus/neonate affected by shoulder presentation - labour/del |
| Q031400 | Fetus or neonate affected by transverse lie in labour/deliv |
| Q031500 | Fetus/neonate affected by face presentation during labour/de |
| Q031600 | Fetus/neonate affected by disproportion during labour/delive |
| Q031611 | Fetus/neonate affected-cephalopelvic disproportion lab./del. |
| Q031z00 | Fetus/neonate affected by malposition/disproportion NOS |
| Q031z11 | Fetus or neonate affected by obstructed labour NEC |
| Q032.00 | Fetus or neonate affected by forceps delivery |
| Q033.00 | Fetus or neonate affected by vacuum extraction delivery |
| Q034.00 | Fetus or neonate affected by caesarean section |
| Q035100 | Fetus/neonate affected by mat analgesic agent in labour/del |
| Q035200 | Fetus/neonate affected by mat general anaesthesia - lab/del |
| Q035300 | Fetus/neonate affected by mat epidural anaesth - labour/del |
| Q035500 | Fetus/neonate affected by maternal pethidine in labour/deliv |
| Q035600 | Fetus/neonate affected by other maternal opiates in lab/del |
| Q036.00 | Fetus or neonate affected by precipitate delivery |
| Q037.00 | Fetus or neonate affected by abnormal uterine contractions |
| Q037.11 | Fetus or neonate affected by hypertonic labour |
| Q037100 | Fetus or neonate affected by hypertonic labour |
| Q037200 | Fetus or neonate affected by hypertonic uterine dysfunction |
| Q037300 | Fetus or neonate affected by uterine inertia or dysfunction |
| Q037312 | Fetus/neonate affected by uterine dysfunction in labour/del |
| Q03y200 | Fetus or neonate affected by induction of labour |
| Q03z.00 | Fetus/neonate affected by complic labour/delivery NOS |
| Q11..00 | Short gestation and unspecified low birthweight problems |
| Q11..11 | Baby born premature |
| Q110.00 | Very premature - less than 1000g or less than 28 weeks |
| Q110.11 | Immature baby |
| Q111.00 | Premature - weight 1000g-2499g or gestation of 28-37weeks |
| Q112.00 | Extreme immaturity |
| Q112.11 | Extreme prematurity - less than 28 weeks |
| Q113.00 | Light for gestational age |
| Q114.00 | Low birthweight |
| Q114000 | Birth weight 1000-2499 g |
| Q115.00 | Extremely low birth weight infant |
| Q115000 | Birth weight 999 g or less |
| Q116.00 | Premature infant 28-37 weeks |
| Q11z.00 | Born premature NOS |
| Q12..00 | Disorders relating to long gestation and high birthweight |
| Q12..11 | Large baby born |
| Q120.00 | Very large baby - weight greater than 4500gm |
| Q121.00 | Other ""large-for-dates"" infant |
| Q122.00 | Postmature infant - greater than 42 weeks gestation, unspec |
| Q12z.00 | Large or postmature infant NOS |
| Q13..00 | Light for gestational age |
| Q2...00 | Birth trauma, asphyxia and hypoxia |
| Q20..00 | Birth trauma |
| Q200.00 | Subdural and cerebral haemorrhage due to birth trauma |
| Q200000 | Cerebral haemorrhage unspecified, due to birth trauma |
| Q200011 | Intracerebral haemorrhage in fetus or newborn |
| Q200012 | Intracranial haemorrhage in fetus or newborn |
| Q200100 | Subdural haemorrhage unspecified, due to birth trauma |
| Q200200 | Local subdural haematoma due to birth trauma |
| Q200300 | Tentorial tear due to birth trauma |
| Q200400 | Brain injury due to birth trauma NOS |
| Q200411 | Cerebral injury due to birth trauma |
| Q200500 | Cerebral haematoma in fetus or newborn |
| Q200511 | Intracerebral haematoma in fetus or newborn |
| Q200600 | Extradural haemorrhage in fetus or newborn |
| Q200700 | Cerebral haemorrhage due to birth injury |
| Q200y00 | Subdural or cerebral haemorrhage due to birth trauma OS |
| Q200z00 | Subdural or cerebral haemorrhage due to birth trauma NOS |
| Q200z11 | Birth brain damage NOS |
| Q201.00 | Scalp injuries due to birth trauma |
| Q201000 | Caput succedaneum due to birth trauma |
| Q201100 | Cephalhaematoma due to birth trauma |
| Q201200 | Vacuum extraction chignon |
| Q201400 | Scalp abrasions due to birth trauma |
| Q201500 | Scalp bruising, due to birth trauma |
| Q201700 | Bruising of scalp due to birth injury |
| Q201y00 | Other specified scalp injury due to birth trauma |
| Q202.00 | Fracture of clavicle due to birth trauma |
| Q203.00 | Other skeleton injury due to birth trauma |
| Q203.11 | Other fractures due to birth trauma |
| Q203.12 | Other birth fracture |
| Q203000 | Fracture of humerus due to birth trauma |
| Q203100 | Fracture of radius or ulna due to birth trauma |
| Q203111 | Birth fracture of radius |
| Q203112 | Birth fracture of ulna |
| Q203200 | Fracture of femur due to birth trauma |
| Q203300 | Fracture of tibia or fibula due to birth trauma |
| Q203400 | Fracture of skull due to birth trauma |
| Q203500 | Birth dislocation of the shoulder |
| Q203600 | Other dislocation or subluxation due to birth trauma |
| Q203y00 | Other specified skeleton injury due to birth trauma |
| Q203y11 | Fracture due to birth trauma NEC |
| Q203y12 | Fracture of nose due to birth trauma |
| Q204.00 | Spine or spinal cord injury due to birth trauma |
| Q204000 | Spine dislocation due to birth trauma |
| Q204z00 | Spine or spinal cord injury due to birth trauma NOS |
| Q205.00 | Facial nerve palsy due to birth trauma |
| Q206.00 | Brachial plexus palsy due to birth trauma |
| Q206000 | Brachial palsy unspecified, due to birth trauma |
| Q206100 | Birth plexus inj - Erb-Duchenne |
| Q206111 | Erb-Duchenne paralysis |
| Q206112 | Erb's palsy |
| Q206200 | Birth plexus injury - Klumpke-Dejerine |
| Q206300 | Birth plexus injury - whole plexus |
| Q206y00 | Other specified brachial plexus palsy due to birth trauma |
| Q206z00 | Brachial plexus palsy due to birth trauma NOS |
| Q207000 | Birth injury to phrenic nerve |
| Q207011 | Phrenic nerve palsy in newborn |
| Q207100 | Cranial nerve injury due to birth trauma |
| Q207200 | Peripheral nerve injury due to birth trauma |
| Q207z00 | Cranial or peripheral nerve palsy due to birth trauma NOS |
| Q208.00 | Cerebral oedema due to birth injury |
| Q20X.00 | Birth injury to central nervous system, unspecified |
| Q20y.00 | Other specified birth trauma |
| Q20y000 | Eye damage due to birth trauma |
| Q20y200 | Testicular haematoma due to birth trauma |
| Q20y300 | Vulval haematoma due to birth trauma |
| Q20y500 | Spleen rupture due to birth trauma |
| Q20y600 | Scalpel wound due to birth trauma |
| Q20y700 | Traumatic glaucoma due to birth trauma |
| Q20y800 | Subconjunctival haemorrhage due to birth trauma |
| Q20y900 | Torticollis due to birth injury |
| Q20yA00 | Birth trauma due to amniocentesis |
| Q20yB00 | Sternomastoid injury due to birth injury |
| Q20yC00 | Birth injury to face |
| Q20yD00 | Subcutaneous fat necrosis due to birth injury |
| Q20yz00 | Other specified birth trauma NOS |
| Q20yz12 | Kidney injury due to birth trauma |
| Q20yz13 | Renal injury due to birth trauma |
| Q20yz14 | Laryngeal injury due to birth trauma |
| Q20yz15 | Toe injury NEC due to birth trauma |
| Q20yz16 | Trunk injury NEC due to birth trauma |
| Q20z.00 | Birth injury NOS |
| Q21..00 | Intrauterine hypoxia and birth asphyxia |
| Q21..11 | Intrauterine hypoxia |
| Q21..12 | Labour fetal anoxia |
| Q212.00 | Liveborn with prelabour fetal distress |
| Q212.11 | Fetal distress before labour - liveborn |
| Q212000 | Liveborn with prelabour abnormal heart beat |
| Q212100 | Liveborn with prelabour hypoxia |
| Q212200 | Liveborn with prelabour meconium in liquor |
| Q212z00 | Liveborn with prelabour fetal distress NOS |
| Q213.00 | Liveborn with labour fetal distress |
| Q213.11 | Fetal distress in labour - liveborn |
| Q213000 | Liveborn with labour abnormal heart beat |
| Q213100 | Liveborn with labour hypoxia |
| Q213z00 | Liveborn with labour fetal distress NOS |
| Q214.00 | Liveborn with fetal distress, unspecified |
| Q214.11 | Fetal distress, unspecified when, liveborn |
| Q214000 | Liveborn with abnormal heart beat, unspecified |
| Q214100 | Liveborn with fetal hypoxia, unspecified |
| Q214200 | Liveborn with meconium liquor, unspecified |
| Q214z00 | Liveborn with unspecified fetal distress NOS |
| Q215.00 | Severe birth asphyxia - apgar score less than 4 at 1 minute |
| Q215.11 | White asphyxia |
| Q216.00 | Mild to moderate birth asphyxia - apgar score 4-7 at 1 min |
| Q216.11 | Blue asphyxia |
| Q21z.00 | Liveborn with birth asphyxia NOS |
| Q21z.11 | Anoxia in newborn NOS |
| Q21z.12 | Hypoxia in newborn NOS |
| Q21z.13 | Birth asphyxia |
| Q2y..00 | Other specified birth trauma, asphyxia or hypoxia |
| Q2z..00 | Birth trauma, asphyxia or hypoxia NOS |
| Q30..00 | Respiratory distress syndrome |
| Q30..11 | Hyaline membrane disease |
| Q30..12 | Pulmonary hypoperfusion syndrome of newborn |
| Q310.00 | Congenital pneumonia |
| Q310000 | Congenital pneumonia due to staphylococcus |
| Q310100 | Congenital pneumonia due to group A haemolytic streptococcus |
| Q310200 | Congenital pneumonia due to group B haemolytic streptococcus |
| Q310300 | Congenital pneumonia due to Escherichia coli |
| Q310400 | Congenital pneumonia due to pseudomonas |
| Q310500 | Congenital pneumonia due to viral agent |
| Q310600 | Congenital pneumonia due to Chlamydia |
| Q310y00 | Other specified congenital pneumonia |
| Q310z00 | Congenital pneumonia NOS |
| Q311.00 | Massive aspiration syndrome |
| Q311000 | Meconium aspiration syndrome |
| Q311100 | Aspiration of liquor or mucus in newborn |
| Q311111 | Aspiration of mucus in newborn |
| Q311112 | Aspiration of liquor in newborn |
| Q311113 | Aspiration of amniotic fluid in newborn |
| Q311200 | Aspiration of blood in newborn |
| Q311300 | Aspiration of vomit in newborn |
| Q311400 | Neonatal aspiration of milk and regurgitated food |
| Q311y00 | Other specified massive aspiration syndrome |
| Q311z00 | Massive aspiration syndrome NOS |
| Q311z11 | Pneumonitis due to fetal aspiration |
| Q312.00 | Perinatal interstitial emphysema and related conditions |
| Q312000 | Perinatal pneumothorax |
| Q312100 | Perinatal pneumomediastinum |
| Q312111 | Perinatal mediastinal emphysema |
| Q312200 | Perinatal pneumopericardium |
| Q312300 | Perinatal interstitial emphysema |
| Q313.00 | Perinatal pulmonary haemorrhage |
| Q313000 | Perinatal lung alveolar haemorrhage |
| Q313100 | Perinatal lung intra-alveolar haemorrhage |
| Q313200 | Perinatal massive pulmonary haemorrhage |
| Q313300 | Perinatal haemoptysis |
| Q313400 | Tracheobronchial haemorrhage origin in the perinatal period |
| Q313z00 | Perinatal pulmonary haemorrhage NOS |
| Q314.00 | Primary atelectasis |
| Q315.00 | Other and unspecified perinatal atelectasis |
| Q316.00 | Newborn transitory tachypnoea |
| Q316.11 | Wet lung syndrome in newborn |
| Q317.00 | Perinatal chronic respiratory disease |
| Q317000 | Perinatal bronchopulmonary dysplasia |
| Q317100 | Prematurity with interstitial pulmonary fibrosis |
| Q317200 | Wilson-Mikity syndrome |
| Q317y00 | Other specified perinatal chronic respiratory disease |
| Q317z00 | Perinatal chronic respiratory disease NOS |
| Q318.00 | Primary sleep apnoea of newborn |
| Q319.00 | Respiratory failure of newborn |
| Q31y.00 | Other perinatal respiratory problems |
| Q31y000 | Perinatal apnoeic spells NOS |
| Q31y100 | Perinatal cyanotic attacks NOS |
| Q31y111 | Cyanotic attacks of the newborn |
| Q31y200 | Perinatal respiratory distress NOS |
| Q31y300 | Perinatal respiratory failure NOS |
| Q31y500 | Neonatal snuffles |
| Q31y511 | Neonatal sniffles |
| Q31y512 | Snuffles |
| Q31y600 | Apnoea of newborn |
| Q31y611 | Neonatal apnoeic attack |
| Q31yz00 | Other perinatal respiratory problems NOS |
| Q31yz11 | Grunting baby |
| Q31z.00 | Perinatal respiratory problems NOS |
| Q400.00 | Congenital rubella |
| Q400.11 | Extended rubella syndrome |
| Q401.00 | Congenital cytomegalovirus infection |
| Q402.00 | Other congenital infections |
| Q402000 | Congenital herpes simplex |
| Q402100 | Congenital listeriosis |
| Q402200 | Congenital malaria |
| Q402300 | Congenital toxoplasmosis |
| Q402312 | Lymphadenopathy due to congenital toxoplasmosis |
| Q402400 | Congenital tuberculosis |
| Q402500 | Congenital falciparum malaria |
| Q402z00 | Other congenital infection NOS |
| Q403.00 | Tetanus neonatorum |
| Q403100 | Tetanus omphalitis |
| Q404.00 | Omphalitis of the newborn |
| Q404.11 | Umbilical stump infection of the newborn |
| Q404000 | Infectious granuloma |
| Q404100 | Omphalitis |
| Q404y00 | Other specified umbilical sepsis |
| Q404z00 | Umbilical sepsis NOS |
| Q405.00 | Neonatal infective mastitis |
| Q406.00 | Neonatal dacryocystitis and conjunctivitis |
| Q406000 | Ophthalmia neonatorum, unspecified |
| Q406100 | Neonatal conjunctivitis |
| Q406200 | Neonatal dacryocystitis |
| Q406300 | Neonatal dacryocystitis or conjunctivitis due to E.Coli |
| Q406311 | Neonatal dacryocystitis due to E.Coli |
| Q406312 | Neonatal conjunctivitis due to E.coli |
| Q406313 | Ophthalmia neonatorum - coliform |
| Q406400 | Neonatal dacryocystitis/conjunctivitis due to staphylococcus |
| Q406411 | Neonatal dacryocystitis due to staphylococcus |
| Q406412 | Neonatal conjunctivitis due to staphylococcus |
| Q406413 | Ophthalmia neonatorum - staphylococcal |
| Q406500 | Neonatal dacryocystitis/conjunctivitis due to other bacteria |
| Q406511 | Neonatal dacryocystitis due to other bacteria |
| Q406512 | Neonatal conjunctivitis due to other bacteria |
| Q406513 | Ophthalmia neonatorum - bacterial NEC |
| Q406600 | Neonatal dacryocystitis or conjunctivitis due to virus |
| Q406611 | Neonatal viral dacryocystitis or conjunctivitis |
| Q406612 | Neonatal dacryocystitis due to virus |
| Q406613 | Neonatal conjunctivitis due to virus |
| Q406614 | Ophthalmia neonatorum - viral |
| Q406700 | Neonatal dacryocystitis or conjunctivitis due to chlamydiae |
| Q406712 | Neonatal conjunctivitis due to chlamydiae |
| Q406713 | Ophthalmia neonatorum - chlamydial |
| Q406800 | Neonatal dacryocystitis/conjunctivitis-other inclusion body |
| Q406811 | Neonatal dacryocystitis due to other inclusion body |
| Q406812 | Neonatal conjunctivitis due to other inclusion body |
| Q406y00 | Other specified neonatal dacryocystitis or conjunctivitis |
| Q406z00 | Neonatal dacryocystitis or conjunctivitis NOS |
| Q407.00 | Neonatal candida infection |
| Q407.11 | Neonatal monilia |
| Q407.12 | Neonatal thrush |
| Q407000 | Neonatal candidiasis of mouth |
| Q407100 | Neonatal candidiasis of perineum |
| Q407200 | Neonatal candidiasis of other skin |
| Q407500 | Neonatal candida septicaemia |
| Q407511 | Neonatal monilial septicaemia |
| Q407y00 | Other specified neonatal candida infection |
| Q407z00 | Neonatal candida infection NOS |
| Q409.00 | Congenital viral hepatitis |
| Q409000 | Congenital hepatitis A infection |
| Q409100 | Congenital hepatitis B infection |
| Q40A.00 | Sepsis of the newborn |
| Q40A000 | Sepsis of newborn due to Staphylococcus aureus |
| Q40A100 | Sepsis of newborn due to Escherichia coli |
| Q40A300 | Perinatal coagulase negative staphylococcus |
| Q40W.00 | Sepsis of newborn due to other+unspecified streptococci |
| Q40X.00 | Congenital viral disease, unspecified |
| Q40y011 | Congenital sepsis NOS |
| Q40y012 | Congenital septicaemia |
| Q40y100 | Neonatal urinary tract infection |
| Q40y200 | Septicaemia of newborn |
| Q41..11 | Neonatal haemorrhage |
| Q411300 | Intraventricular haemorrhage due to birth injury |
| Q412000 | Subarachnoid haemorrhage due to birth injury |
| Q413.00 | Umbilical haemorrhage after birth |
| Q413200 | Massive umbilical haemorrhage |
| Q413z00 | Umbilical haemorrhage after birth NOS |
| Q414300 | Neonatal vaginal haemorrhage |
| Q41y.00 | Other fetal and newborn haemorrhage |
| Q41y200 | Neonatal haematemesis |
| Q41y300 | Neonatal melaena |
| Q41y400 | Neonatal rectal haemorrhage |
| Q42..11 | Isoimmunisation of newborn |
| Q420.00 | Haemolytic disease due to rhesus isoimmunisation |
| Q420.11 | Erythroblastosis fetalis |
| Q420.12 | Rhesus isoimmunisation of the newborn |
| Q421.11 | ABO isoimmunisation of the newborn |
| Q425.00 | Late anaemia of newborn due to isoimmunisation |
| Q430000 | Neonatal jaundice + glucose-6-phosphate dehydrogenase defic. |
| Q432.00 | Preterm delivery associated jaundice |
| Q433.00 | Other neonatal jaundice - delayed conjugation other cause |
| Q433000 | Delayed conjugation causing neonatal jaundice, unspecified |
| Q433200 | Breast feeding inhibitors causing neonatal jaundice |
| Q433300 | Lucy - Driscoll syndrome |
| Q433500 | Neonatal jaundice with Dubin-Johnson syndrome |
| Q433600 | Neonatal jaundice with Gilbert's syndrome |
| Q433700 | Neonatal jaundice with congenital hypothyroidism |
| Q433800 | Neonatal jaundice with porphyria |
| Q433A00 | Neonatal jaundice from breast milk inhibitor |
| Q433y00 | Delayed conjugation causing neonatal jaundice OS |
| Q433y11 | Neonatal jaundice - deficiency enzyme for bilirubin conjug. |
| Q433z00 | Delayed conjugation causing neonatal jaundice NOS |
| Q436200 | Newborn physiological jaundice NOS |
| Q437.00 | Kernicterus not due to isoimmunisation |
| Q437000 | Bilirubin encephalopathy |
| Q437z00 | Kernicterus of newborn NOS |
| Q44..00 | Perinatal endocrine and metabolic problems |
| Q440.00 | Infant of a diabetic mother"" syndrome |
| Q441.00 | Neonatal diabetes mellitus |
| Q442.00 | Neonatal myasthenia gravis |
| Q443.00 | Neonatal thyrotoxicosis |
| Q444.00 | Neonatal hypocalcaemia |
| Q444.12 | Neonatal hypoparathroidism |
| Q444011 | Neonatal cows' milk hypocalcaemia |
| Q444111 | Hypocalcaemic tetany in newborn |
| Q444z00 | Neonatal hypocalcaemia NOS |
| Q445.00 | Neonatal hypomagnesaemia |
| Q446.00 | Other transitory neonatal electrolyte disturbance |
| Q446000 | Neonatal dehydration |
| Q446100 | Transitory neonatal hypernatraemia |
| Q446200 | Transitory neonatal hyponatraemia |
| Q446300 | Transitory neonatal hyperkalaemia |
| Q446400 | Transitory neonatal hypokalaemia |
| Q446500 | Disturbances of sodium balance of newborn |
| Q446600 | Disturbances of potassium balance of newborn |
| Q446y00 | Other specified transitory neonatal electrolyte disturbance |
| Q446z00 | Transitory neonatal electrolyte disturbance NOS |
| Q447.00 | Neonatal hypoglycaemia |
| Q447000 | Iatrogenic neonatal hypoglycaemia |
| Q448.00 | Newborn late metabolic acidosis |
| Q449.00 | Neonatal tetany without calcium or magnesium deficiency |
| Q44B.00 | Syndrome of infant of mother with gestational diabetes |
| Q44T.00 | Transitory metabolic disturbance of newborn, unspecified |
| Q44U.00 | Transitory neonatal endocrine disorder, unspecified |
| Q44V.00 | Neonatal goitre, not elsewhere classified |
| Q44W.00 | Transitory neonatl disord calcium and magnes metab uns |
| Q44y000 | Transitory neonatal tyrosinaemia |
| Q44y100 | Transitory metabolic disturbance-infant pre-diabetic mother |
| Q44z.00 | Perinatal endocrine or metabolic problem NOS |
| Q45..00 | Fetal and newborn blood disorders |
| Q450.00 | Haemorrhagic disease of the newborn |
| Q450.11 | Vitamin K deficiency of the newborn |
| Q451.00 | Transient neonatal thrombocytopenia |
| Q451y00 | Other specified transient neonatal thrombocytopenia |
| Q451y11 | Neonatal thrombocytopenia due to platelet alloimmunisation |
| Q451z00 | Transient neonatal thrombocytopenia NOS |
| Q452.00 | Newborn disseminated intravascular coagulation |
| Q454.00 | Polycythaemia neonatorum |
| Q454000 | Polycythaemia due to donor twin transfusion |
| Q454z00 | Polycythaemia neonatorum NOS |
| Q455.00 | Congenital anaemia |
| Q455000 | Congenital anaemia from fetal blood loss |
| Q456.00 | Anaemia of prematurity |
| Q457.00 | Transient neonatal neutropenia |
| Q457000 | Neonatal isoimmune neutropenia |
| Q457z00 | Transient neonatal neutropenia NOS |
| Q45y.00 | Other specified transient neonatal blood disorder |
| Q45z.00 | Fetal or newborn blood disorder NOS |
| Q460.00 | Meconium ileus |
| Q462.00 | Newborn swallowing maternal blood - haematemesis/melaena |
| Q463.00 | Transitory ileus of newborn |
| Q466.00 | Other meconium obstruction |
| Q466000 | Meconium plug syndrome |
| Q466100 | Congenital faecoliths causing obstruction |
| Q466z00 | Meconium obstruction NOS |
| Q466z11 | Delayed passage of meconium NOS |
| Q46X.00 | Intestinal obstruction of newborn, unspecified |
| Q46y000 | Neonatal peritonitis NOS |
| Q46y100 | Neonatal diarrhoea |
| Q46yz00 | Other perinatal digestive system disorder NOS |
| Q470.00 | Idiopathic hydrops fetalis |
| Q471.00 | Sclerema neonatorum |
| Q472.00 | Newborn cold injury syndrome |
| Q473.00 | Other hypothermia of newborn |
| Q474000 | Newborn dehydration fever |
| Q474100 | Newborn environmental pyrexia |
| Q474111 | Newborn environmental hyperthermia |
| Q474200 | Hyperthermia in newborn, unspecified |
| Q474211 | Thermal injury in newborn NEC |
| Q474300 | Transitory fever of newborn |
| Q474z00 | Newborn temperature regulation disorder NOS |
| Q475.00 | Oedema of newborn unspecified |
| Q476.00 | Congenital hydrocele |
| Q476.11 | Patent processus vaginalis |
| Q477.00 | Newborn breast engorgement |
| Q478.00 | Neonatal erythema toxicum |
| Q47y000 | Urticaria neonatorum |
| Q47y200 | Neonatal skin infection |
| Q47yy11 | Erythroderma neonatorum |
| Q47yy12 | Panniculitis in newborn |
| Q480.00 | Convulsions in newborn |
| Q480.11 | Fits in newborn |
| Q480.12 | Seizures in newborn |
| Q481.00 | Newborn cerebral irritability, unspecified |
| Q482.00 | Central nervous system dysfunction in newborn NOS |
| Q482000 | Newborn cerebral depression |
| Q482100 | Coma in newborn |
| Q482y00 | Other newborn abnormal cerebral signs |
| Q482z00 | Central nervous system dysfunction in newborn NOS |
| Q483.00 | Feeding problems in newborn |
| Q483000 | Newborn feeding problem, unspecified |
| Q483100 | Newborn regurgitation of food |
| Q483200 | Slow feeding in newborn |
| Q483300 | Vomiting in newborn |
| Q483400 | Rumination in newborn |
| Q483500 | Underfeeding in newborn |
| Q483600 | Overfeeding in newborn |
| Q483700 | Difficulty in feeding at breast |
| Q483z00 | Newborn feeding problem NOS |
| Q483z11 | Bottle feeding problem in the newborn |
| Q483z12 | Breast feeding problem in the newborn |
| Q484.00 | Newborn drug reaction and intoxication |
| Q484000 | Grey syndrome of newborn |
| Q485.00 | Newborn drug withdrawal syndrome |
| Q485000 | Neonat withdrawal symptom from mat use of drug of addiction |
| Q485100 | Withdrawal symptoms from therapeutic use of drugs in newborn |
| Q485200 | Neonatal Abstinence Syndrome |
| Q487.00 | Umbilical polyp of newborn |
| Q488.00 | Neonatal cerebral ischaemia |
| Q489.00 | Acquired periventricular cysts of newborn |
| Q48A.00 | Neonatal cerebral leukomalacia |
| Q48B.00 | Jittery baby |
| Q48C.00 | Neonatal hypotension |
| Q48E.00 | Periventricular leucomalacia |
| Q48E.11 | Periventricular leukomalacia |
| Q48y000 | Congenital renal failure |
| Q48y100 | Congenital cardiac failure |
| Q48y200 | Congenital hypotonia |
| Q48y211 | Floppy infant |
| Q48y212 | Floppy baby |
| Q48y300 | Congenital hypertonia |
| Q48y400 | Wide cranial sutures |
| Q48y411 | Neonatal ""craniotabes"" |
| Q48y500 | Megalencephaly |
| Q48y600 | Early neonatal death |
| Q48y700 | Late neonatal death |
| Q48yz00 | Other perinatal condition NOS |
| Q48yz11 | Congenital hepatic fibrosis |
| Q49..00 | Cardiovascular disorders originating in the perinatal period |
| Q490.00 | Neonatal cardiac failure |
| Q491.00 | Neonatal cardiac dysrhythmia |
| Q492.00 | Neonatal hypertension |
| Q493.00 | Persistent fetal circulation |
| Q494.00 | Transient myocardial ischaemia of newborn |
| Q49X.00 | Cardiovasc disord origin in the perinat period, unspecif |
| Q4z..11 | Infant death |
| Q4z..12 | Neonatal death |
| Q4z..13 | Newborn death |
| Qyu1000 | [X]Other low birth weight |
| Qyu1100 | [X]Other preterm infants |
| Qyu2200 | [X]Other birth injuries to scalp |
| Qyu3000 | [X]Other respiratory distress of newborn |
| Qyu3500 | [X]Oth pulmonary haemorrhages originating/perinatal period |
| Qyu3600 | [X]Other chronic resp diseases originating/perinatal period |
| Qyu3800 | [X]Other apnoea of newborn |
| Qyu3900 | [X]Other specified respiratory conditions of newborn |
| Qyu3A00 | [X]Oth cardiovascular disorders originating/perinatal period |
| Qyu4100 | [X]Sepsis/newborn due to other+unspecified staphylococcus |
| Qyu4200 | [X]Other bacterial sepsis of newborn |
| Qyu4800 | [X]Sepsis of newborn due to other+unspecified streptococci |
| Qyu5800 | [X]Neonat jaun due/drg,toxn transmit frm mother/given newbrn |
| Qyu5900 | [X]Neonatal jaundice due/other specifd excessive haemolysis |
| Qyu5A00 | [X]Neonatal jaundice from other+unspcf hepatocellular damage |
| Qyu5B00 | [X]Neonatal jaundice from other specified causes |
| Qyu5C00 | [X]Other congenital anaemias, not elsewhere classified |
| Qyu6000 | [X]Other neonatal hypoglycaemia |
| Qyu6C00 | [X]Transitory metabolic disturbance of newborn, unspecified |
| Qyu8000 | [X]Other hypothermia of newborn |
| Qyu8400 | [X]Hypothermia of newborn, unspecified |
| QyuA000 | [X]Oth specified disturbances of cerebral status of newborn |
| QyuA200 | [X]Other disorders of muscle tone of newborn |
| QyuA400 | [X]Disorder of muscle tone of newborn, unspecified |
| Z241.00 | Labour established |
| Z241100 | Onset of labour induced |
| Z243.00 | Observation of first stage of labour |
| Z243100 | First stage of labour established |
| Z243200 | First stage of labour not established |
| Z243300 | Progress of labour - first stage |
| Z243400 | Rapid first stage of labour |
| Z243411 | Rapid progress in first stage of labour |
| Z243500 | Normal length of first stage of labour |
| Z243600 | Slow progress in first stage of labour |
| Z243800 | First stage of labour problem |
| Z244100 | Observation of duration of labour |
| Z244300 | Short duration of labour |
| Z244400 | Late onset of labour |
| Z244411 | Postmature labour |
| Z244500 | Relation of onset of labour to due date |
| Z245.00 | Observation of blood loss in labour |
| Z245100 | Maternal blood loss minimal |
| Z245200 | Maternal blood loss within normal limits |
| Z245300 | Maternal blood loss moderate |
| Z245400 | Maternal blood loss heavy |
| Z246.00 | Observation of measures of labour |
| Z246100 | Duration of labour |
| Z246200 | Onset of labour first stage |
| Z246211 | Start of labour |
| Z246300 | Onset of contractions |
| Z246311 | Onset of labour pains |
| Z246400 | Time contractions became regular |
| Z246600 | Time rupture of membranes detected |
| Z246611 | Time waters ruptured |
| Z246700 | Onset of second stage of labour |
| Z246900 | Duration of second stage of labour |
| Z246A00 | Total duration of labour |
| Z246B00 | Estimated maternal blood loss |
| Z246B11 | EBL - Estimated maternal blood loss |
| Z247100 | Expulsion of IUCD during third stage of labour |
| Z248.00 | Normal labour |
| Z249.00 | Labour problem |
| Z25..00 | Delivery observations |
| Z251.00 | Mother delivered |
| Z253.12 | Rate of delivery |
| Z253100 | Slow rate of delivery |
| Z253200 | Rapid rate of delivery |
| Z253211 | Precipitate delivery |
| Z253300 | Normal rate of delivery |
| Z254.00 | Observation of pattern of delivery |
| Z254100 | Deliveries by forceps - delivered |
| Z254200 | Delivered by low forceps delivery |
| Z254300 | Delivered by mid-cavity forceps delivery |
| Z254400 | Deliveries by breech extraction |
| Z254500 | Delivered by caesarean section - pregnancy at term |
| Z254600 | Deliv caes following prev caes |
| Z254700 | Deliveries by vacuum extractor |
| Z254800 | Deliveries by spontaneous breech delivery |
| Z254900 | Vaginal delivery |
| Z254A00 | Abnormal delivery |
| Z254B00 | Brow delivery |
| Z254C00 | Face delivery |
| Z254D00 | Face to pubes birth |
| Z254E00 | Multiple birth |
| Z254E11 | Multiple birth delivery |
| Z255.00 | Observation of second stage of labour |
| Z255300 | Progess of second stage of labour |
| Z255400 | Rapid second stage of labour |
| Z255500 | Progressing well in second stage |
| Z255700 | Failure to progress in second stage of labour |
| Z255711 | No progress with delivery |
| Z255800 | Second stage of labour problem |
| Z255A00 | Observation of delivery push in labour |
| Z255B00 | Desire to push |
| Z255D00 | Ability to push in labour |
| Z255F00 | Not pushing well in labour |
| Z255J00 | Pushing involuntarily in labour |
| Z256200 | Prolonged third stage of labour |
| Z256500 | Delayed expulsion of placenta |
| Z257.00 | Delivery normal |
| Z257.11 | Normal delivery |
| Z257.12 | Spontaneous vaginal delivery |
| Z257.13 | SVD - Spontaneous vaginal delivery |
| Z257.14 | FTND - Full term normal delivery |
| Z257.15 | ND - Normal delivery |
| Z257100 | Spontaneous vertex delivery |
| Z258.00 | Delivery problem |
| Z262600 | Complete placenta at delivery |
| Z262700 | Incomplete placenta at delivery |
| Z262711 | Incomplete delivery of placenta |
| Z265900 | Umbilical cord not around baby's neck at delivery |
| Z29..00 | Postnatal examination observations |
| ZV24.00 | [V]Postpartum care and examination |
| ZV24.11 | [V]Postnatal care and examination |
| ZV24000 | [V]Examination immediately after delivery |
| ZV24100 | [V]Examination of Lactating mother |
| ZV24200 | [V]Routine postpartum follow-up |
| ZV24y00 | [V]Other specified postpartum care and examination |
| ZV24z00 | [V]Unspecified postpartum care and examination |
| ZV27.11 | [V]Live birth |
| ZV27000 | [V]Single live birth |
| ZV27200 | [V]Twins, both live born |
| ZV27300 | [V]Twins, one live born and one stillborn |
| ZV27500 | [V]Other multiple birth, all live born |
| ZV29.00 | [V]Level of neonatal care |
| ZV29.11 | [V] Level of care administered to neonates |
| ZV29000 | [V]Normal level of neonatal care |
| ZV29011 | [V] Normal care administered to neonates |
| ZV29100 | [V]Newborn receiving special care |
| ZV29111 | [V]Neonatal care in SCBU |
| ZV29200 | [V]Newborn receiving intensive care |
| ZV29211 | [V]Neonatal care on ITU |
| ZV29212 | [V]Neonatal care on NNU |
| ZV29300 | [V]Level 2 intensive care (high dep int care) admin neonate |
| ZV3..00 | [V]Healthy liveborn infants according to type of birth |
| ZV30.00 | [V]Singleton |
| ZV30000 | [V]Singleton, born in hospital |
| ZV30100 | [V]Singleton, born before admission to hospital |
| ZV30z00 | [V]Singleton NOS |
| ZV31.00 | [V]Twin, mate live born |
| ZV31000 | [V]Twin, born in hospital, mate live born |
| ZV31200 | [V]Twin, not hospitalised, mate live born |
| ZV31z00 | [V]Twin, mate liveborn, NOS |
| ZV32.00 | [V]Twin, mate stillborn |
| ZV32000 | [V]Twin, born in hospital, mate stillborn |
| ZV32z00 | [V]Twin, mate stillborn, NOS |
| ZV33.00 | [V]Twin, unspecified |
| ZV33000 | [V]Twin, unspecified, born in hospital |
| ZV33z00 | [V]Twin, unspecified, NOS |
| ZV36100 | [V]Other multiple birth, before hospital, mates live+still |
| ZV3yz00 | [V]Other multiple birth, unspecified, NOS |
| ZV3z.00 | [V]Unspecified birth |
| ZV3z000 | [V]Unspecified birth, born in hospital |
| ZV3zz00 | [V]Unspecified birth, NOS |

1. Entity types are record type identifiers within Vision that control the entry of specific clinical data such as birth details or antenatal visit information, and also control the way in which test results are recorded. [↑](#footnote-ref-2)
