# Supplementary material for: Methods to generate and validate a Pregnancy Register in the UK Clinical Practice Research Datalink primary care database
Source: Pharmacoepidemiol Drug Saf. 2019 Jun 13;28(7):923–33. doi: 10.1002/pds.4811 (PMC6618019; doi:10.1002/pds.4811)
Supplement: Supplementary file 8 — Data S8: Supporting Information [file PDS-28-923-s008.docx]

**The Clinical Practice Research Datalink (CPRD) Pregnancy Register Algorithm**

Four key components are involved in generating the CPRD Pregnancy Register:

1. Compiling and categorising comprehensive lists of pregnancy codes;
2. Extracting records of these codes and associated data fields among female patients in the CPRD GOLD database, and data on gestational age and timing of birth from their infants (identified in the CPRD GOLD Mother-Baby link);
3. Developing and applying hierarchical rules to determine pregnancy episodes from these data;
4. Creating a summary record for each identified pregnancy episode.

**1. Pregnancy code list compilation**

In CPRD GOLD, clinical-care events are coded using Read codes and Entity types. Each code is accompanied by an event date, and some (Entity) codes have additional information associated with the event recorded in other data fields. We compiled a list of more than 4,000 pregnancy-related Read and Entity codes. We flagged each code according to 21 non-mutually exclusive categories relating to the antenatal period, the pregnancy outcome, the postnatal period, additional features of the pregnancy, and pregnancy dates (see Figure 1 below). Our full categorised code lists are provided in S3 and S4.

|  | **Pregnancy code categories** | |
| --- | --- | --- |
|  | Antenatal | |
| antenatal period | Late pregnancy (≤3 weeks before delivery) | |
|  | Third trimester | |
|  |  | |
|  | Delivery | |
|  | Stillbirth | |
|  | Ectopic | |
|  | TOP (termination of pregnancy) | |
| pregnancy outcome | Miscarriage | |
|  | Probable TOP | |
|  | Molar pregnancy | |
|  | Unspecified pregnancy loss | |
|  | Blighted ovum | |
|  |  | |
| postnatal period | Postnatal (≤8 weeks after delivery) | |
|  | Other postnatal | |
|  |  | |
|  | Preterm | |
| additional features | Post-term | |
|  | Multiple | |
|  |  | |
|  | LMP (last menstrual period - 1^st^ day) | |
| pregnancy dates | EDD (estimated date of delivery) | |
|  | EDC (estimated date of conception) | |
|  |  | |
| other | Pregnancy related (timing uncertain) | |
| Figure 1. Categories of Pregnancy Read and Entity codes. | |  |

**2. Data extraction**

The pregnancy codes are used to identify and extract all pregnancy-related records from Clinical, Referral, Test, and “Additional Clinical Details” files among female patients from CPRD GOLD practices, aged between 11 and 49 years, who conform to the standard patient acceptability criteria.

For patients who are identified in the CPRD GOLD Mother-Baby link, additional data are extracted from linked infant records in the first year of life (see Table 1).

Table 1. Data extracted from infants in the CPRD GOLD Mother-Baby link.

| **Linked infant data** | **Pregnancy code categories used** |
| --- | --- |
| Records specifying the number of days/weeks after delivery | Postnatal (≤8 weeks after delivery) |
| Year and month of birth | Not applicable |
| Earliest consultation with the GP | Not applicable |
| Records indicating gestational age at birth | Preterm, Post-term, Delivery |
| Records indicating a multiple pregnancy | Multiple |

**3. Overall approach**

The algorithm uses these data in a series of Stages (1-8, described below) to delineate pregnancy episodes, assigning to each one an estimated start date (defined as the date of the first day of the woman’s last menstrual period (LMP), in keeping with UK clinical practice), an estimated end date, and an outcome (when recorded). S2-Table shows how the algorithm uses the pregnancy codes and associated data fields, for each category of code.

The algorithm begins by identifying and grouping together records relating to the outcome of pregnancy and estimating the pregnancy end date. The end date is then used as a point of reference from which the start of pregnancy is estimated, in combination with all available data on gestational age, and pregnancy dates: LMP, estimated date of delivery (EDD), and estimated date of conception (EDC). Antenatal records occurring between the estimated start and end dates of pregnancy, and records relating to the outcome, are assigned to the pregnancy. The algorithm determines additional characteristics, including the type of pregnancy outcome and whether it is a preterm, post-term or multiple pregnancy, from information within these assigned records and from linked infant records (live births only).

***Stage 1. Identify pregnancy outcome records.***

Maternal records relating to pregnancy outcomes (deliveries and early pregnancy losses), and maternal and infant records relating to the early postnatal period (up to 8 weeks postpartum) are identified from the extracted data.

Possible end-of-pregnancy dates are derived from each record, as follows:

- When the code specifies a number of days or weeks after delivery (e.g. “Maternal P/N 6 week exam”), the specified duration is subtracted from the event date.
- When the code relates to the postnatal (or perinatal) period but does not specify a number of days/weeks after delivery (e.g. “Postnatal care”), 14 days are subtracted from the event date (7 days, if perinatal).
- For Entity type 114 (Pregnancy outcome), 2 days are subtracted from the Discharge date.
- For all other delivery records and early pregnancy loss records, the event date is used.

***Stage 2. Determine the date of a woman’s first pregnancy outcome.***

Records relating to deliveries (including postnatal records) are considered separately to those relating to early pregnancy losses.

The date of each woman’s first delivery (or loss) is estimated by taking the earliest of:

1. All possible end-of-pregnancy dates derived in Stage 1; and, for deliveries with a linked infant identified in the CPRD Mother-Baby link:
2. The infant’s date of birth, initially taking the last day of the month of birth (MOB), and
3. The earliest infant consultation.

When ii) is the earliest, the 15^th^ day of the infant’s MOB is used to estimate the delivery date. When i) or iii) precede the infant’s MOB by up to 6 weeks, the 1^st^ day of the infant’s MOB is used to estimate the delivery date.

***Stage 3. Combine records relating to a woman’s first pregnancy outcome and determine the type of outcome.***

Additional records relating to a woman’s first pregnancy outcome are identified and assigned to the outcome, as follows:

For deliveries: all delivery-related records (identified in Stage 1) which occur <25 weeks after the estimated delivery date are assigned to that delivery episode. Our choice of 25 weeks allows for a minimum separation of 25 weeks between successive deliveries, assuming a minimum gestational age of 24 weeks for a completed pregnancy ending in live birth or stillbirth, and the earliest ovulation occurring 3 weeks after delivery.

Deliveries are characterised as preterm, post-term, multiple, or stillbirth, using the following information:

- Code categories of the assigned delivery-related records;
- Preterm, Post-term or Multiple pregnancy codes in maternal records <24 weeks before the estimated date of delivery;
- Additional data from linked infant records (see Data extraction, above).

For early pregnancy losses: all early pregnancy loss records (identified in Stage 1) which occur <8 weeks after the estimated pregnancy loss date are assigned to that pregnancy loss episode. Our choice of 8 weeks allows for a minimum separation of 8 weeks between successive early pregnancy losses, assuming a minimum gestational age of 7 weeks for a documented early pregnancy loss, and the earliest ovulation occurring 2 weeks after a miscarriage. The type of early pregnancy loss is determined by the presence of an assigned code in the following categories, ranked in descending order of priority (see Table 2).

Table 2. Hierarchy for determining the type of early pregnancy loss.

| **Level in hierarchy** | **Early pregnancy loss code category** |
| --- | --- |
| i | Ectopic |
| ii | TOP (termination of pregnancy) |
| iii | Miscarriage |
| iv | Probable TOP |
| v | Molar pregnancy |
| vi | Unspecified pregnancy loss |
| vii | Blighted ovum |

The hierarchy enables the classification of early pregnancy loss episodes with assigned codes from different categories. Thus, for any given episode, an Ectopic code takes precedence over all other assigned early pregnancy loss codes. Blighted ovum is lowest in the hierarchy to allow for the possibility that this outcome can co-exist in a viable pregnancy.

***Stage 4. Date and characterise each successive pregnancy outcome.***

Stages 2 & 3 are repeated to identify, date and characterise women’s subsequent pregnancy outcomes sequentially, using delivery-related records occurring ≥25 weeks after the previous delivery, and early pregnancy loss records occurring ≥8 weeks after the previous early pregnancy loss.

***Stage 5. Estimate the start of each pregnancy.***

Records relating to the timing of the start of pregnancy are identified from the extracted data. These records comprise the code categories shown in Table 3, in descending order of priority.

Table 3. Hierarchy of information used to estimate the start of pregnancy.

| **Level in hierarchy** | **Code categories for estimating the start of pregnancy** |
| --- | --- |
| i | EDD* |
| ii | EDC* |
| iii | LMP* |
| iv | Antenatal codes specifying gestational age |
| v | Delivery codes specifying gestational age at birth (assigned to the delivery in Stages 3 & 4, or extracted from linked infants’ records). |

* EDD, EDC and LMP records based on Read codes are only used when the event date (the date associated with the event, as entered by the GP) does not equal the system date (the date the event was entered into the Vision software system).

Possible pregnancy start dates are calculated from each record, as follows:

1. For EDD records, 280 days are subtracted from the event date (for Read codes) or from the “Expected date of delivery” in the relevant data field (for Entity types).
2. For EDC records, 14 days are subtracted from the event date.
3. For LMP records, the event date is used.
4. For Antenatal records, the specified number of completed weeks’ gestation is subtracted from the event date, e.g. 128 days for a record of “A/N 16 week examination”. When a range is specified rather than an exact number of weeks, the middle of the range is used (for ranges of ≤14 days). When the range exceeds 14 days (e.g. “Antenatal ultrasound scan at 17-22 weeks”), the record is deemed too imprecise to calculate LMP.
5. For Delivery records, the specified gestational age at birth is subtracted from the estimated date of delivery (determined in Stages 2 & 4), e.g. 252 days is subtracted for a record of “Baby premature 36 weeks”. As for Antenatal records, when a range of gestational weeks is specified, the middle of the range is used (for ranges of ≤14 days). However, when the range exceeds 14 days (e.g. “Baby extremely prem.28-32 weeks”), the upper limit of the range is used (in this example, 32 weeks) as the durations of most preterm pregnancies are assumed to be closer to the upper limit.

For each patient, a mapping of all possible pregnancy start dates to pregnancy outcomes (identified in Stages 2-4) is performed, allowing up to 41 weeks between the start and end of pregnancy for preterm or multiple deliveries, 46 weeks for all other deliveries, 12 weeks for ectopic pregnancies and 24 weeks for all other early pregnancy losses. For deliveries, start dates derived from all five code categories are used, whereas only those derived from categories i-iv are used for early pregnancy losses.

Multiple mappings (possible start dates) per pregnancy outcome are handled as follows:

- Within each of the first four categories (i, ii, iii, iv), a pregnancy start date derived from an antenatal ultrasound scan takes precedence over all other estimates, otherwise the record with an event date closest to the pregnancy outcome date is used to derive the start date. Within category v, the start date derived from the most precise record of gestational age at birth is selected.
- For pregnancy outcomes with potential start dates derived from more than one category, the estimate derived from the record highest in the hierarchy is selected.

If no pregnancy start date can be derived for a pregnancy outcome, the start date is imputed by subtracting a default duration from the estimated outcome date, according the type of pregnancy outcome (see Table 4).

Table 4. Default durations applied by the algorithm, by type of pregnancy outcome.

| **Pregnancy outcome** | **Default duration (completed weeks)** |
| --- | --- |
| Preterm delivery* | 36 |
| Post-term delivery* | 41 |
| Multiple delivery† | 37 |
| All other deliveries* | 40 |
| Ectopic pregnancy | 9 |
| All other early pregnancy losses | 12 |

* live birth or stillbirth

† multiple only, i.e. no evidence for preterm or post-term

***Stage 6. Adjust the pregnancy start and end dates (if necessary).***

Adjustments to estimated pregnancy start and end dates are made in two phases. The first phase applies to deliveries only; the second phase applies to both deliveries and early pregnancy losses.

First, each delivery identified by the algorithm in the preceding Stages is assessed for plausibility of gestational age. When the estimated pregnancy duration (days difference between the estimated start and end dates) falls outside plausible limits for the type of delivery, adjustments are made to the estimated start or end dates, by subtracting the minimum number of days required to generate a plausible gestational age. Deliveries with and without assigned records of gestational age at birth are dealt with separately, as follows:

For deliveries with assigned records of gestational age at birth: the estimated duration is compared with the upper and lower limits (or exact number) of gestational weeks specified in the record. When the estimated duration is less than the specified number of weeks (or lower limit), the start date is adjusted to generate the specified (minimum) gestational age. When the estimated duration exceeds the specified number of weeks (or upper limit), the end date is adjusted to generate the specified (maximum) gestational age.

For deliveries with no assigned records of gestational age at birth: the end dates are adjusted when necessary to generate the maximum plausible duration for the type of pregnancy (42 weeks for term/post-term deliveries, 36 weeks plus 6 days for preterm deliveries, 37 weeks for multiple deliveries).

Second, all Antenatal records are identified from the extracted pregnancy data and their proximity to the estimated pregnancy start dates is assessed. For each pregnancy identified by the algorithm, any Antenatal records occurring in the 4 weeks before the estimated start date are assumed to correspond to that pregnancy; hence, the estimated start date is assumed to be late. In this situation, the algorithm subtracts 4 weeks from the earliest of these Antenatal records to generate a revised pregnancy start date (assuming the earliest indication of pregnancy to be 4 weeks after the 1^st^ day of a woman’s LMP). Similar adjustment is made to the pregnancy end date to preserve the estimated gestational age of the pregnancy.

***Stage 7. Assign antenatal records to each pregnancy.***

All Antenatal records occurring between the estimated start and end dates of pregnancy are assigned to the pregnancy. Additionally, because some Antenatal codes may be recorded retrospectively after a pregnancy has ended (for example, a record of pre-eclampsia or other pregnancy complication), any such codes identified in the 4 weeks after the pregnancy end date are assumed to correspond to that pregnancy and hence are assigned to it (deliveries only). These codes are a subset of the Antenatal codes, flagged in the Read code list (S3-Table) as “Postdel_antenatal”.

***Stage 8. Identify additional pregnancies with no recorded outcome.***

Additional pregnancies (those with no recorded outcome) are identified using all remaining unassigned Antenatal records (those not yet assigned to a previously identified pregnancy).

First, “Late pregnancy” codes are used. These are a subset of the Antenatal codes relating to the final stage of pregnancy (<3 weeks before delivery). Successive Late pregnancy records are grouped together (those occurring <25 weeks after the initial record) and characterised in the same way as delivery-related records (Stage 3). One week is added to the event date of the latest of these grouped records to obtain an estimated pregnancy end date. Subsequent pregnancies based on Late pregnancy codes are identified, dated and characterised sequentially, using unassigned Late pregnancy records occurring ≥25 weeks after the previous episode. Pregnancy start dates are determined in the same way as for other pregnancies (using the hierarchy described in Stage 5). Adjustments to start and end dates are made (as per Stage 6) and antenatal records are assigned to each pregnancy (as per Stage 7).

Second, “Third trimester” codes are used. These are a subset of the Antenatal codes relating to gestational week 27 onwards. Successive Third trimester records relating to the same pregnancy are grouped together (those occurring <16 weeks after the initial record) and characterised (as per Stage 3). As for Late pregnancy records, one week is added to the event date of the latest record in an episode to obtain an estimated pregnancy end date. Subsequent pregnancies based on Third trimester codes are identified, dated and characterised sequentially, using unassigned Third trimester records occurring ≥28 weeks after the previous episode. Our choice of 28 weeks allows for a minimum separation of 28 weeks between a delivery and the start of the third trimester for a subsequent pregnancy. Each pregnancy’s start date is estimated using the Third trimester record which specifies the longest gestational age (by subtracting the specified duration from the record’s event date). The same methods of date adjustment and antenatal record assignment are applied as for Late pregnancy episodes.

Third, any remaining unassigned Antenatal records are combined into pregnancy episodes, each comprising successive Antenatal records <6 weeks apart. Unlike pregnancies based on Late pregnancy or Third trimester records (which are assumed to culminate in delivery), the outcomes of these pregnancies are not known. However, the pregnancy start dates are determined using gestational age specified in Antenatal records (when available), by subtracting the specified duration from the record’s event date. When more than one record per episode specifies gestational age, the latest record is selected. When no information on gestational age is available, the pregnancy start date is imputed by subtracting 4 weeks from the earliest Antenatal record in the episode.

**4. Generating summary Pregnancy records**

A summary record for each identified pregnancy episode is generated, comprising the estimated start, end and trimester dates, the type of data used for date estimation, and whether adjustments to the dates were made; the pregnancy outcome; the linked infant identifier (live births only); the gestational age; additional characteristics (evidence for preterm, post-term, or multiple pregnancy); and whether the pregnancy episode overlaps with another identified episode. Summary pregnancy records for each patient are listed chronologically in a single file (the Pregnancy Register). The full list of patient-level and pregnancy-level variables provided in the Pregnancy Register is shown in S1-Table.
